# Age Differences in the Discrimination and Level‐Specific Risk of Emergency Severity Index Triage for Critical Outcomes in the Oldest‐Old

**DOI:** 10.1111/ggi.70900

**Published:** 2026-11-08

**Authors:** Hoang Le Huy Nguyen

**Affiliations:** ^1^ School of Mathematics and Leeds Institute for Data Analytics University of Leeds Leeds UK

**Keywords:** aged 80 and over, emergency service hospital, risk assessment, ROC curve, triage

## Abstract

**Aim:**

To compare Emergency Severity Index (ESI) discrimination, level‐specific risk, and under‐triage for a critical outcome between emergency department (ED) stays aged 80 years and older and 18–64 years.

**Methods:**

Retrospective cohort of 418 025 ED stays of 201 214 adults with an ESI level at one US academic center, 2011–2019 (MIMIC‐IV‐ED). The critical outcome was death in the ED or hospital, or intensive care admission within 24 h. Measures were the ESI C statistic, transportability of each level's risk from 18 to 64 years, under‐triage odds (ESI 3–5 in an outcome stay), and differences after case‐mix stratification and reweighting.

**Results:**

The C statistic was 0.818 at 18–64 and 0.754 at 80 and older (difference, −0.065; 95% CI, −0.072 to −0.058). The difference was −0.040 (95% CI, −0.048 to −0.033) pooled within arrival‐mode strata and −0.058 (95% CI, −0.071 to −0.046) after case‐mix reweighting. At 80 and older, observed risk was 1.96 times that carried by the same levels at 18 to 64. Specificity of ESI 1 or 2 was 48.8% versus 69.0%. Under‐triage occurred in 11.1% of outcome stays at 80 and older and 14.0% at 18–64 (adjusted odds ratio, 0.81; 95% CI, 0.73–0.90), but in 1.42 versus 0.55 per 100 stays.

**Conclusions:**

At 80 years and older, ESI levels ranked critical‐outcome risk less well, a difference smaller within arrival‐mode strata, and levels 1–3 carried more risk than in younger adults. Age‐specific triage rules need prospective study.

## Graphical abstract

In 418 025 emergency department stays, Emergency Severity Index levels ranked critical‐outcome risk less well at 80 years and older than at 18–64 (C statistic 0.754 vs. 0.818), less so within arrival‐mode strata, and levels 1–3 carried more risk in the oldest‐old.

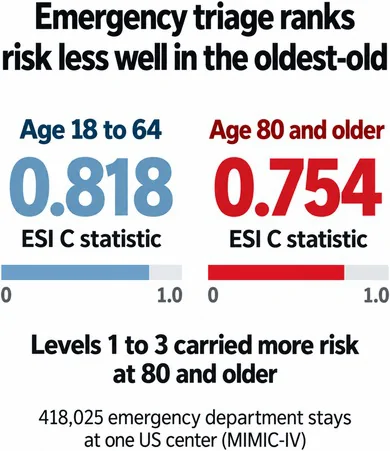

## 1 Introduction

Adults aged 80 years and older are a growing share of emergency department (ED) visits and have a higher risk of death or early intensive care than younger adults, yet one Emergency Severity Index (ESI) governs how quickly they are seen. The ESI was validated in patients of all ages, but older patients often present with blunted vital signs and nonspecific complaints that its decision points do not weight for age [1, 2]. An age‐specific threshold or a geriatric flag at triage is worth testing only if the ESI levels rank and quantify risk less well in the oldest‐old.

In single‐center studies of patients aged 65 and older, ESI Level 1 missed most of those receiving an immediate life‐saving intervention (519–782 patients) [2, 3], and the ESI predicted hospitalization only moderately (929 patients; area under the curve 0.77) [1]; none had a younger comparison group. The one study with a comparison group enrolled 2370 older and 3141 younger patients in Finland over 1 month and examined 3‐day mortality [4]. A trauma‐center comparison found no age difference [5], and a systematic review reported no pooled estimate by age [6]. No study has estimated ESI discrimination for death or early intensive care across age bands in a large US cohort. Nor has any study examined whether the risk carried by each level in younger patients holds in older patients (transportability), or whether an age gap remains after adjustment for triage vital signs and chief complaint.

This is a retrospective cohort study of ED stays at one US academic center from 2011 to 2019, linked to hospital and intensive care records. It compared ESI discrimination and the transportability of level‐specific risk for death or intensive care admission within 24 h between stays at 80 years and older and at 18–64 years. It also examined whether under‐triage of stays with this critical outcome differed by age after adjustment for triage vital signs and chief complaint.

## 2 Methods

### 2.1 Study Design and Oversight

This retrospective cohort study used adult ED stays at Beth Israel Deaconess Medical Center, Boston, from 2011 to 2019. Data came from the MIMIC‐IV‐ED database (version 2.2) [7], linked to MIMIC‐IV (version 3.1) [8, 9] for hospital and intensive care unit (ICU) records. It evaluated an existing instrument, the ESI [10], and developed no model. Reporting followed STROBE, RECORD, and TRIPOD+AI for an existing instrument [11, 12, 13]. The analysis plan was dated before analysis but not registered. The author vouches for the accuracy and completeness of the data and analyses. Generative AI tools were used to improve language, clarity, and readability. The author reviewed and approved the final manuscript and takes full responsibility for its content.

### 2.2 Participants

All ED stays were eligible. Stays were excluded, in order, if age at arrival could not be derived or was under 18, or if no ESI level was recorded. Age at arrival was derived from the MIMIC‐IV anchor age. MIMIC‐IV records ages above 89 years as 91, so exact age is unknown in the 90 and older band; no stay is placed in the wrong band (eTable 1). Age bands were 18–64 years (reference), 65–74, 75–79, 80–84, 85–89, and 90 and older; the oldest‐old were 80 and older. All stays of each patient were analyzed; a patient's first stay was the index stay.

### 2.3 Measurements

The triage nurse assigned the ESI level, from 1 (highest acuity) to 5. Triage vital signs were heart rate, systolic blood pressure, respiratory rate, oxygen saturation, and temperature; values outside plausibility ranges, set before analysis, were treated as missing (eTable 2). The free‐text chief complaint was mapped by keyword to 16 categories (eMethods). Arrival mode and sex were recorded; a stay linked to a hospital admission counted as admitted.

### 2.4 Outcomes

The critical outcome was death in the ED or during the linked hospitalization, or first ICU arrival more than 0 and at most 24 h after ED arrival. Stays not admitted could contribute only ED death. Under‐triage was ESI Level 3–5 in a stay with a critical outcome. The primary estimands were the discrimination (C statistic) of the ESI level for the critical outcome and the transportability of the risk carried by each level, each estimated by band and contrasted between 80 years and older and 18–64 years. The secondary estimand was the odds of under‐triage in each older band relative to 18–64 years, before and after adjustment.

### 2.5 Statistical Analysis

Discrimination was the nonparametric C statistic of the ESI level within each band: the probability that a stay with the critical outcome had been assigned a higher‐acuity level than a stay without it, with ties counted one half. Its variance summed the placement‐value influence function within patient to allow for repeat stays [14]. Variances of band differences summed influence contributions within patient across both bands, because 2794 patients had stays at both 18–64 and 65–74 years. Because the ESI assigns levels, not probabilities, transportability was assessed by applying the observed risk at each level at 18–64 years (levels 4 and 5 combined) to each older band as its expected risk [15]. Agreement was summarized by a transport intercept (logistic regression with the logit of the expected risk as offset), a transport slope (regression on that logit), and observed to expected ratios (Table 2 gives their interpretation).

Under‐triage was analyzed among stays with a critical outcome by logistic regression on age band, with variances cluster‐robust on patient. Models were unadjusted and adjusted for the five vital signs (restricted cubic splines, three knots) [16] and chief‐complaint category. Age band was coded with six levels and, in a second model, three (18–64, 65–79, 80 and older). Missing vital signs, assumed missing at random given covariates, were imputed by predictive mean matching (20 data sets, 10 iterations) [17], and estimates were combined by Rubin's rules [18].

Additional analyses covered index stays, complete cases, an age band by ESI level interaction, alternative outcome windows, fuller adjustment, and anchor‐year groups (patient‐level de‐identified calendar windows); standard‐error ratios served as a clustering diagnostic. Analyses specified after the primary results were known covered outcome components and absolute performance. They also covered discrimination within arrival‐mode and chief‐complaint strata (pooled by inverse variance) and after inverse‐odds reweighting on pre‐triage covariates, as well as bounds for stays without an ESI level and for transfers. Complaint‐mapping agreement was described (eMethods). Confidence intervals for secondary and additional analyses were not adjusted for multiplicity and do not support definitive inference. Analyses used R version 4.6.1 with the data.table, sandwich [19], mice [17], splines, and geepack [20] packages and random seed 20260913.

## 3 Results

### 3.1 Participants

Of 425 087 ED stays of 205 504 patients, 76 were excluded because age could not be derived and 6986 because no ESI level was recorded (Figure 1), leaving 418 025 stays of 201 214 patients. Stays without an ESI level made up 2.1% of stays at 80 years and older and 1.5% at 18–64 years. Of these stays, 63.3% and 42.3% had the critical outcome, 9.7% and 14.5% of the band's outcome events (eTable 1). The critical outcome occurred in 25 407 stays (6.1%); 23 788 of these were ICU admissions within 24 h. Among 49 731 stays at 80 and older, the critical outcome occurred in 6338 (12.7%), versus 11 209 of 287 140 (3.9%) at 18–64 (Table 1).

**FIGURE 1:**
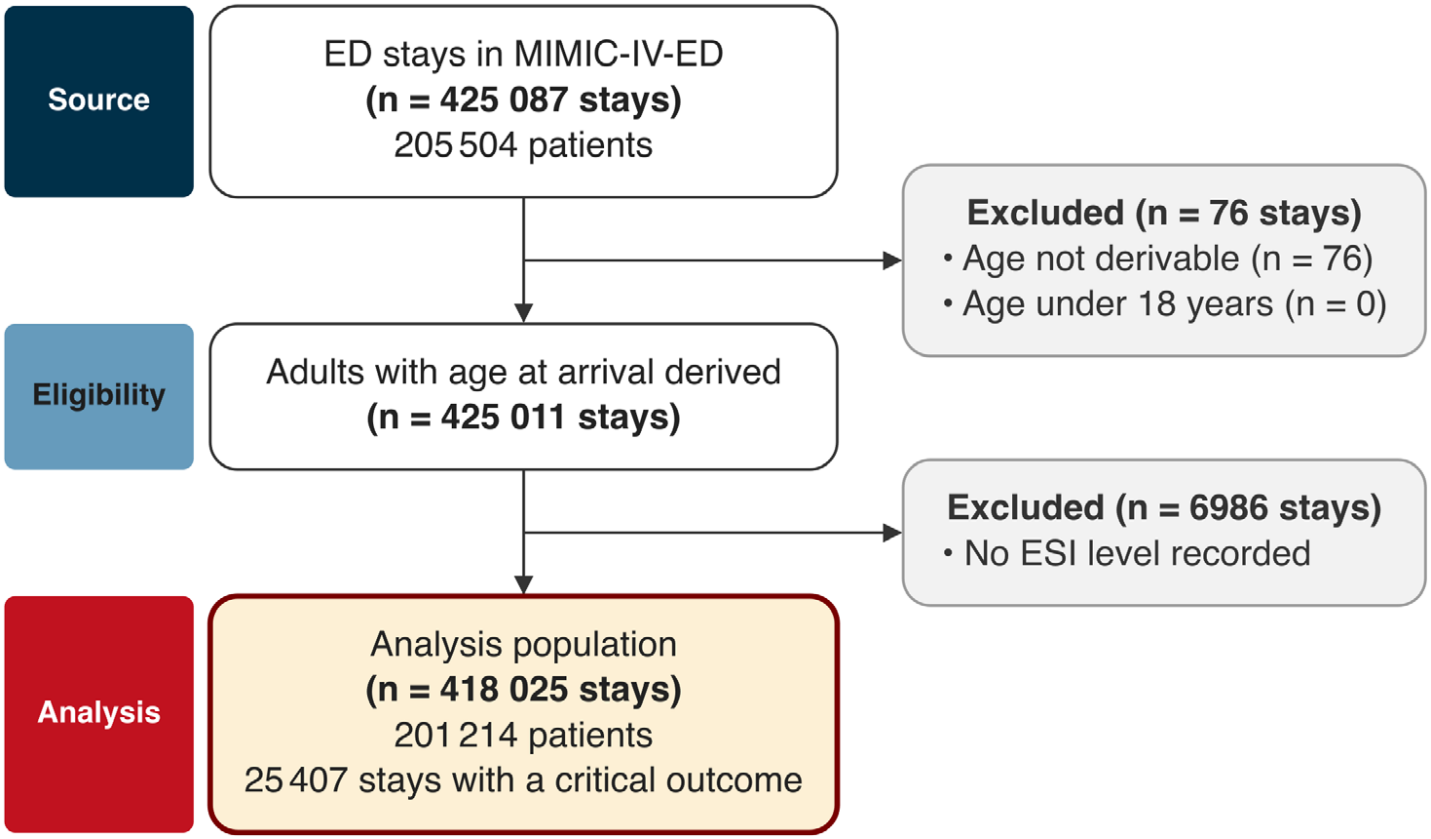
Participant flow. Selection of emergency department (ED) stays of adults with a recorded Emergency Severity Index (ESI) level at one US academic center, 2011 to 2019, with the number of stays remaining after each exclusion and of patients at the start and in the analysis population. The two age criteria share one exclusion box; no stay was excluded for age under 18 years.

**TABLE 1:** Characteristics of the analysis population, overall and by age band.

Characteristic	Overall	18–64 year	65–74 year	75–79 year	80–84 year	85–89 year	90 year and older	80 year and older
Stays, No.	418 025	287 140	58 711	22 443	19 687	16 121	13 923	49 731
Patients, No.	201 214	147 894	27 574	11 372	9820	7786	6132	21 100
Age, median (IQR), year	53 (35–69)	43 (29–54)	69 (67–72)	77 (76–78)	82 (81–83)	87 (86–88)	91 (91–91)	86 (83–90)
Sex, No. (%)								
Female	226 976 (54.3)	154 689 (53.9)	30 267 (51.6)	12 004 (53.5)	11 004 (55.9)	9658 (59.9)	9354 (67.2)	30 016 (60.4)
Male	191 049 (45.7)	132 451 (46.1)	28 444 (48.4)	10 439 (46.5)	8683 (44.1)	6463 (40.1)	4569 (32.8)	19 715 (39.6)
Arrival mode, No. (%)								
Walk‐in	251 420 (60.1)	192 333 (67.0)	31 368 (53.4)	10 473 (46.7)	8007 (40.7)	5485 (34.0)	3754 (27.0)	17 246 (34.7)
Ambulance	150 401 (36.0)	83 277 (29.0)	25 283 (43.1)	11 124 (49.6)	10 983 (55.8)	10 047 (62.3)	9687 (69.6)	30 717 (61.8)
Other or unknown	16 204 (3.9)	11 530 (4.0)	2060 (3.5)	846 (3.8)	697 (3.5)	589 (3.7)	482 (3.5)	1768 (3.6)
ESI level, No. (%)								
Level 1	24 014 (5.7)	13 070 (4.6)	4337 (7.4)	1800 (8.0)	1817 (9.2)	1520 (9.4)	1470 (10.6)	4807 (9.7)
Level 2	139 390 (33.3)	82 091 (28.6)	24 320 (41.4)	9927 (44.2)	9015 (45.8)	7499 (46.5)	6538 (47.0)	23 052 (46.4)
Level 3	225 024 (53.8)	165 700 (57.7)	27 968 (47.6)	10 162 (45.3)	8518 (43.3)	6890 (42.7)	5786 (41.6)	21 194 (42.6)
Level 4	28 497 (6.8)	25 297 (8.8)	2004 (3.4)	535 (2.4)	331 (1.7)	203 (1.3)	127 (0.9)	661 (1.3)
Level 5	1100 (0.3)	982 (0.3)	82 (0.1)	19 (0.1)	6 (< 0.1)	9 (0.1)	2 (< 0.1)	17 (< 0.1)
Admitted to hospital, No. (%)	197 799 (47.3)	110 093 (38.3)	36 214 (61.7)	15 040 (67.0)	14 034 (71.3)	11 834 (73.4)	10 584 (76.0)	36 452 (73.3)
Critical outcome, No. (%)^a^	25 407 (6.1)	11 209 (3.9)	5418 (9.2)	2442 (10.9)	2399 (12.2)	2051 (12.7)	1888 (13.6)	6338 (12.7)
Death in ED	197 (< 0.1)	66 (< 0.1)	33 (0.1)	15 (0.1)	23 (0.1)	21 (0.1)	39 (0.3)	83 (0.2)
Death in hospital	3841 (0.9)	1028 (0.4)	842 (1.4)	451 (2.0)	498 (2.5)	485 (3.0)	537 (3.9)	1520 (3.1)
ICU admission within 24 h	23 788 (5.7)	10 796 (3.8)	5056 (8.6)	2280 (10.2)	2200 (11.2)	1834 (11.4)	1622 (11.6)	5656 (11.4)
Under‐triage among critical outcomes, No. (%)^b^	3147 (12.4)	1565 (14.0)	621 (11.5)	256 (10.5)	253 (10.5)	239 (11.7)	213 (11.3)	705 (11.1)

*Note:* Chief‐complaint category and triage vital signs by age band are in eTable 2.

Abbreviations: ED, emergency department; ESI, Emergency Severity Index; ICU, intensive care unit; IQR, interquartile range.

^a^ Death in the ED or the linked hospitalization, or ICU admission within 24 h of ED arrival. A stay can have more than one component, so the components sum to more than the total.

^b^ Under‐triage among critical outcomes is the percentage of stays with a critical outcome that had been assigned ESI Level 3 to 5 at triage.

In the 220 226 stays (52.7%) without a linked admission, the in‐hospital death flag was absent and was counted as no event. After implausible values were set to missing, heart rate was missing in 10 313 stays (2.5%) and in 4660 (18.3%) of stays with a critical outcome (eTable 2). The imputation produced no warnings.

### 3.2 Discrimination and Level‐Specific Risk

The C statistic of the ESI level was 0.818 (95% CI, 0.814–0.822) at 18–64 years and 0.754 (95% CI, 0.748–0.759) at 80 years and older (difference, −0.065; 95% CI, −0.072 to −0.058). It was lower than at 18–64 years in every older band (Table 2). With the risk of each level at 18–64 years as expected risk, the transport intercept at 80 and older was 0.85 (95% CI, 0.82–0.88) and the slope 0.88 (95% CI, 0.85–0.91). Observed risk was 1.96 times the expected risk overall, 1.64 times at ESI 1 (46.7% vs. 28.4%), 2.03 times at ESI 2, and 3.54 times at ESI 3 (Table 2; Figure 2; eTable 3). At ESI 4 and 5, the reference band had only 12 events in 26 279 stays, so ratios at these levels are not interpreted. All models converged.

**TABLE 2:** Discrimination and transportability of level‐specific risk of the ESI Level for the critical outcome by age band.

Age band	Critical outcomes, No.^a^	C statistic (95% CI)	Difference versus 18–64 year (95% CI)^b^	Transport intercept (95% CI)^c^	Transport slope (95% CI)^c^	Observed to expected ratio^c^
18–64 year	11 209	0.818 (0.814–0.822)		0.00 (−0.03 to 0.03)	1.00 (0.98–1.02)	1.00
65–74 year	5418	0.774 (0.768–0.780)	−0.044 (−0.051 to −0.037)	0.62 (0.59–0.66)	0.93 (0.90–0.96)	1.67
75–79 year	2442	0.770 (0.761–0.779)	−0.048 (−0.058 to −0.039)	0.76 (0.71–0.81)	0.95 (0.90–0.99)	1.84
80–84 year	2399	0.760 (0.751–0.769)	−0.058 (−0.068 to −0.048)	0.82 (0.77–0.87)	0.91 (0.86–0.95)	1.92
85–89 year	2051	0.749 (0.739–0.759)	−0.069 (−0.080 to −0.058)	0.86 (0.80–0.91)	0.87 (0.82–0.91)	1.98
90 year and older	1888	0.749 (0.738–0.760)	−0.069 (−0.081 to −0.057)	0.88 (0.83–0.94)	0.86 (0.81–0.91)	2.00
80 year and older	6338	0.754 (0.748–0.759)	−0.065 (−0.072 to −0.058)	0.85 (0.82–0.88)	0.88 (0.85–0.91)	1.96

Abbreviations: CI, confidence interval; ESI, Emergency Severity Index.

^a^ Stays and patients in each band are in Table 1.

^b^ Differences use a variance that sums influence contributions within patient across both bands, because 2794 patients have stays in both the 65 to 74 and the 18 to 64 bands. No other band shares patients with 18–64 years.

^c^ The expected risk is the observed risk at each ESI level (1, 2, 3, and 4 and 5 combined) at 18 to 64 years, applied to each older band; the reference band's intercept near 0 and slope near 1 reflect that role. An intercept above 0 means higher observed risk on average than expected; a slope below 1 means that risk differs less between levels than in the reference band. The observed to expected ratio is events divided by the sum of expected risks.

**FIGURE 2:**
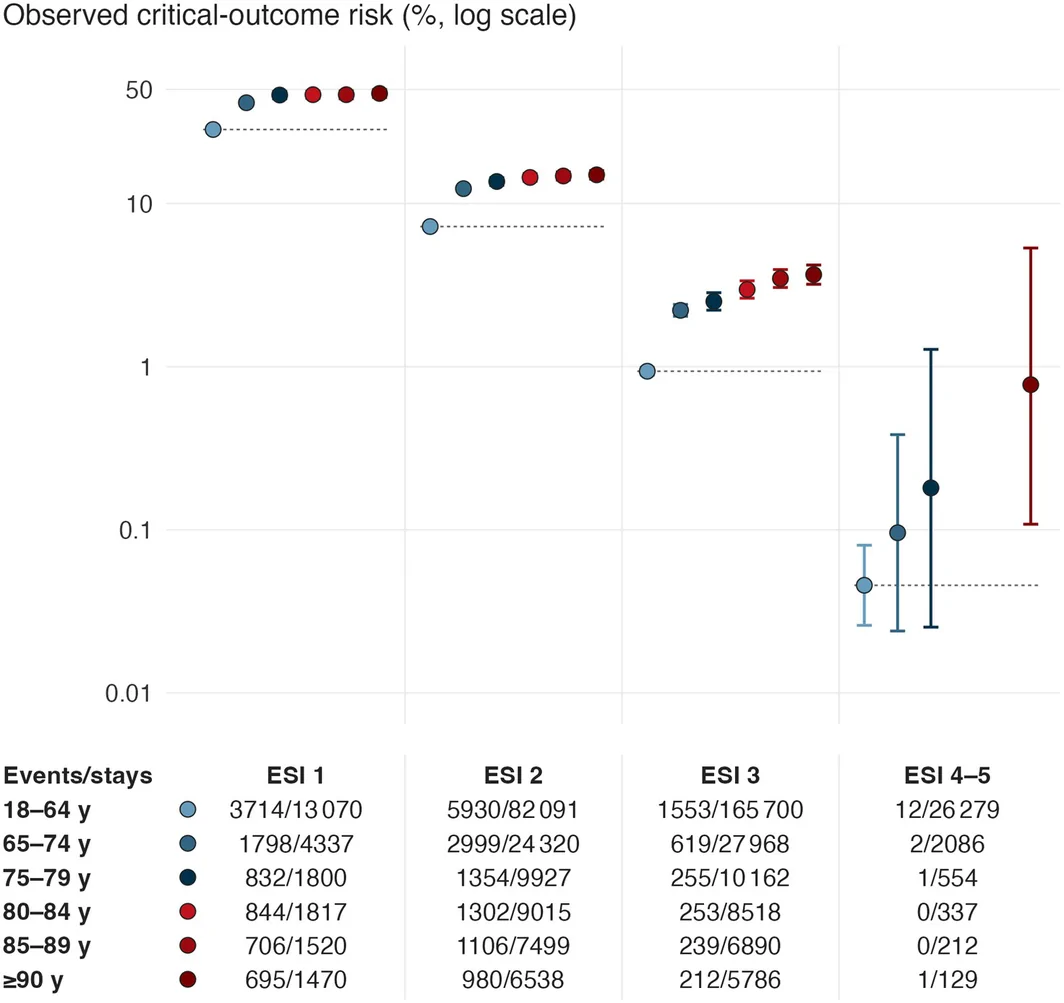
Observed risk of the critical outcome by Emergency Severity Index (ESI) level and age band. Points show observed risk, and bars show 95% confidence intervals that account for repeat stays by the same patient; the vertical axis is logarithmic. The dashed line in each ESI group marks the risk at 18 to 64 years, which was applied as the expected risk in older bands, so points above it show excess risk. The table below the plot gives events/stays for each cell. No critical outcome was observed at ESI 4‐5 in stays aged 80 to 84 years (0/337) or 85 to 89 years (0/212). These cells have counts only, because the patient‐cluster‐robust logit interval used for the other cells cannot be computed when no events occur. Zero observed events does not mean zero risk.

The age difference was present for each outcome component (eTable 4): −0.061 (95% CI, −0.068 to −0.054) for ICU admission within 24 h, −0.082 (95% CI, −0.098 to −0.065) for death in the ED or hospital, and −0.087 (95% CI, −0.107 to −0.068) for death within 7 days of arrival.

With ESI 1 or 2 as a positive test, sensitivity was 88.9% (95% CI, 88.1–89.6) at 80 and older and 86.0% (95% CI, 85.3–86.7) at 18–64. Specificity was 48.8% (95% CI, 48.2–49.4) and 69.0% (95% CI, 68.8–69.3), and positive predictive value was 20.2% and 10.1% (Table 3).

**TABLE 3:** Under‐triage and absolute performance of the ESI level by age band.

Panel A. Odds ratios for under‐triage by age band relative to 18–64 year				
Age band	Unadjusted OR (95% CI)	Adjusted OR with multiple imputation (95% CI)^a^	Complete case OR (95% CI)^b^	Fuller adjustment OR (95% CI)^c^
Six age bands relative to 18–64 year				
65–74 year	0.80 (0.72–0.88)	0.81 (0.73–0.91)	0.78 (0.69–0.87)	0.80 (0.72–0.90)
75–79 year	0.72 (0.63–0.83)	0.73 (0.62–0.84)	0.72 (0.62–0.84)	0.71 (0.61–0.83)
80–84 year	0.73 (0.63–0.84)	0.74 (0.63–0.86)	0.70 (0.60–0.82)	0.70 (0.60–0.82)
85–89 year	0.81 (0.70–0.94)	0.85 (0.73–1.00)	0.81 (0.69–0.95)	0.81 (0.69–0.94)
90 year and older	0.78 (0.67–0.92)	0.87 (0.73–1.02)	0.86 (0.72–1.02)	0.82 (0.69–0.97)
Two age bands relative to 18 to 64 year				
65–79 year	0.77 (0.71–0.85)	0.79 (0.71–0.87)	0.76 (0.69–0.84)	0.77 (0.70–0.85)
80 year and older	0.77 (0.70–0.85)	0.81 (0.73–0.90)	0.78 (0.70–0.87)	0.77 (0.69–0.85)

Panel B. Absolute performance with ESI Level 1 or 2 as the positive test^d^					
Age band	Sensitivity, % (95% CI)	Specificity, % (95% CI)	PPV, % (95% CI)	NPV, % (95% CI)	Outcome stays at ESI 3–5 per 100 stays (95% CI)
18–64 year	86.0 (85.3–86.7)	69.0 (68.8–69.3)	10.1 (9.9–10.4)	99.2 (99.1–99.2)	0.55 (0.52–0.58)
65–74 year	88.5 (87.6–89.4)	55.2 (54.6–55.8)	16.7 (16.2–17.3)	97.9 (97.8–98.1)	1.06 (0.97–1.15)
75–79 year	89.5 (88.2–90.7)	52.3 (51.4–53.2)	18.6 (17.9–19.5)	97.6 (97.3–97.9)	1.14 (1.01–1.29)
80–84 year	89.5 (88.1–90.6)	49.8 (48.8–50.7)	19.8 (19.0–20.6)	97.1 (96.8–97.5)	1.29 (1.14–1.45)
85–89 year	88.3 (86.9–89.7)	48.8 (47.8–49.7)	20.1 (19.2–21.0)	96.6 (96.2–97.0)	1.48 (1.31–1.68)
90 year and older	88.7 (87.2–90.1)	47.4 (46.2–48.5)	20.9 (20.0–21.9)	96.4 (95.9–96.9)	1.53 (1.34–1.75)
80 year and older	88.9 (88.1–89.6)	48.8 (48.2–49.4)	20.2 (19.7–20.7)	96.8 (96.5–97.0)	1.42 (1.32–1.53)

Abbreviations: CI, confidence interval; ESI, Emergency Severity Index; NPV, negative predictive value; OR, odds ratio; PPV, positive predictive value.

^a^ Adjusted for triage vital signs and chief‐complaint category, combined across 20 imputed data sets.

^b^ Restricted to stays with complete triage vital signs and chief‐complaint category.

^c^ Adjusted for triage vital signs, chief‐complaint category, arrival mode, sex, and anchor‐year group (MIMIC‐IV patient‐level de‐identified calendar windows, not the encounter year).

^d^ Intervals are patient‐cluster‐robust. The false‐negative rate is 100 minus sensitivity. eTable 5 gives the false‐negative rate and the ESI 1–3 threshold.

### 3.3 Case Mix

Within arrival‐mode strata, the C statistic difference was −0.016 (95% CI, −0.025 to −0.007) among ambulance arrivals and −0.084 (95% CI, −0.102 to −0.067) among walk‐in arrivals; pooled, it was −0.040 (95% CI, −0.048 to −0.033). Pooled within the 14 chief‐complaint strata with at least 20 events in each group, it was −0.069 (95% CI, −0.076 to −0.062). With stays at 80 and older reweighted to the covariate distribution of younger stays (effective sample size 10 389), the difference was −0.058 (95% CI, −0.071 to −0.046) (eTable 6).

### 3.4 Under‐Triage

Among stays with a critical outcome, 1565 of 11 209 (14.0%) at 18–64 and 705 of 6338 (11.1%) at 80 and older had been assigned ESI Level 3–5. The odds ratio for under‐triage at 80 and older versus 18–64 was 0.77 (95% CI, 0.70–0.85) unadjusted and 0.81 (95% CI, 0.73–0.90) after adjustment for vital signs and chief‐complaint category (Table 3). All fits converged. Per 100 stays overall in each band, critical‐outcome stays at ESI 3–5 numbered 1.42 (95% CI, 1.32–1.53) at 80 and older and 0.55 (95% CI, 0.52–0.58) at 18–64.

### 3.5 Additional Analyses

In the complete‐case analysis (18 606 of 25 407 outcome stays), the adjusted odds ratio at 80 and older was 0.78 (95% CI, 0.70–0.87). With arrival mode, sex, and anchor‐year group added, it was 0.77 (95% CI, 0.69–0.85) (Table 3). In index stays (201 214), the C statistic difference at 80 and older was −0.085 (95% CI, −0.094 to −0.076) and the adjusted under‐triage odds ratio was 0.86 (95% CI, 0.74–1.01) (eTable 7). An age band by ESI level interaction test gave a Wald χ^2^_20_ of 1075.6 (*p* < 0.001). With alternative outcome windows, the difference at 80 and older was −0.069 for ICU admission within 12 h, −0.064 within 48 h, and −0.065 with 7‐day death added (eTable 8; eFigure 1). Across anchor‐year groups, the C statistic rose from 0.795 to 0.834 at 18 to 64 and ranged from 0.744 to 0.755 at 80 and older (eTable 9). Clustering diagnostics are in eTable 10.

When all stays without an ESI level were placed at ESI 1, the difference was −0.067 (95% CI, −0.073 to −0.060). Over every possible assignment of these stays, it lay between −0.167 and 0.082. Counting all 438 transfers at 80 and older and 1499 at 18–64 as having the outcome gave −0.063 (95% CI, −0.071 to −0.056). Reassigning transfers in 10% steps within each ESI level kept the difference between −0.089 and −0.037 (eTable 11). Among stays with a recorded diagnosis, the chief‐complaint category agreed with the first‐listed ED diagnosis in 49.1%–67.5% of trauma, chest pain, and respiratory complaints at 80 and older and in 55.7%–70.4% at 18–64 (eTable 12).

## 4 Discussion

In this single‐center cohort, ESI levels ranked critical‐outcome risk less well at 80 years and older than at 18–64, and the shortfall was present in every older band, from −0.044 at 65 to 74 to −0.069 at both 85 to 89 and 90 and older. The gap was smaller within arrival‐mode strata: it was small among ambulance arrivals, who were most of the oldest‐old, and larger among walk‐in arrivals. It persisted within complaint strata and after reweighting. Levels 1–3 carried more risk at 80 and older than at 18–64. Among stays that had the outcome, the oldest‐old were less often assigned ESI 3–5 after adjustment.

### 4.1 Earlier Studies

Baumann and Strout reported an area under the curve of 0.77 for hospitalization at 65 and older, close to the present value at 80 and older for a different outcome [1]. Kemp and colleagues, in a Finnish cohort over 1 month, found no significant difference in ESI accuracy between patients under and over 65 for any outcome [4]. Unlike that study, the present cohort was far larger, used a fixed critical outcome, and cut age at 80 rather than 65. Because the difference was smaller at 65–74 than at 80 and older, a single cut at 65 that pools these bands may dilute it. With the Japanese and Korean scales, derived from the Canadian Triage and Acuity Scale, discrimination for intensive care admission also fell with age [21, 22]. Between studies of different triage systems, case‐mix severity has shown no clear association with performance [23]; within this cohort, the age difference varied with arrival mode.

### 4.2 Ranking and Absolute Risk

Discrimination and level‐specific risk describe how all five levels order and quantify risk. Under‐triage among critical‐outcome stays describes only one end of the scale. Earlier findings on sensitivity concerned the top level: the ESI missed most older patients receiving an immediate life‐saving intervention [2, 3], and triage scales identify fewer than four in five critically ill patients as high acuity [6].

In the present cohort, sensitivity of ESI 1 or 2 was slightly higher at 80 and older, but specificity was much lower, because many more older stays without the outcome were assigned high acuity. At 80 and older, where the outcome occurred in about one stay in eight, observed risk exceeded the risk each level carried in younger patients. The relative excess was largest at Level 3 and the absolute excess at Level 1. Because the outcome was more common, critical‐outcome stays at levels 3–5 were more frequent per 100 stays than at 18–64, although their share of outcome stays was lower. Ranking and absolute risk are separate properties of any risk instrument [24]; an instrument that ranks well but misstates risk can still mislead decisions [25].

### 4.3 Possible Explanations

This design cannot establish why the levels rank risk less well with age; the explanations below are inferences. First, case mix may contribute, although arrival mode partly reflects acuity itself. Second, the triage inputs may carry less information. Acute illness in older adults presents atypically, with blunted fever, absent classic symptoms, and altered mental status in place of localizing complaints [26, 27, 28], and the rise in respiratory rate with hypoxemia weakens with age [29]. Thresholds written for the general population may therefore separate high from low risk less clearly at 80.

Third, the way levels are assigned may differ with age: in chart reviews of older patients, under‐triage was traced to neglect of high‐risk situations and misread vital signs [2] and to departures from the ESI's own rules [3]. Fourth, intensive care admission depends on goals of care and comorbidity, which triage does not capture. The difference was also present for death alone, with larger point estimates and overlapping intervals; this does not exclude a contribution of admission decisions.

### 4.4 Implications

The finding does not show that an age‐specific threshold or a geriatric flag would improve outcomes. In Finland, age adjustment of two triage methods improved prediction of some outcomes but not others [30]. Geriatric screening at triage has been implemented in routine care and prospectively validated [31, 32], and in one prospective study of patients over 65, a frailty tool outperformed the ESI for intensive care admission [33]. Older adults present at higher acuity and are admitted more often [34, 35], and stays at 80 and older were 11.9% of this cohort. A retrospective single‐center analysis does not by itself support changing practice; whether an age‐specific rule could help needs prospective study.

### 4.5 Limitations

The composite outcome missed deaths within 7 days after stays without a linked admission: 39 (0.35% of composite events) at 18–64 years and 54 (0.85%) at 80 and older (eTable 13). Intensive care admission after transfer could not be observed. Because the completeness of death recording after such stays is unknown, the size and direction of any resulting bias are unknown. The bounds for stays without an ESI level crossed zero, so the result cannot be shown to be robust to their exclusion, although placing them all at ESI 1 left it unchanged. The cohort is one academic center with time‐shifted dates, so the size of the gap elsewhere is untested. Under‐triage odds were adjusted only for triage vital signs and chief complaint. The keyword mapping of chief complaint agreed only moderately with ED diagnoses, and functional status, comorbidity, and goals of care were unmeasured. No chart review of missed cases was possible, so instrument failure and assignment failure cannot be separated.

### 4.6 Conclusions

At 80 years and older, ESI levels ranked critical‐outcome risk less well than at 18–64, a difference smaller within arrival‐mode strata, and levels 1–3 carried more risk than in younger adults. Age‐specific triage rules need prospective study.

## Author Contributions

**Hoang Le Huy Nguyen:** conceptualization, methodology, formal analysis, investigation, writing – original draft, writing – review and editing.

## Funding

The author has nothing to report.

## Ethics Statement

MIMIC‐IV and MIMIC‐IV‐ED were each approved by the Institutional Review Board (IRB) of Beth Israel Deaconess Medical Center (MIMIC‐IV‐ED under IRB #2001P001699), which granted a waiver of informed consent and approved the data‐sharing initiative for this retrospective, de‐identified electronic health record data. This study was conducted in accordance with the Declaration of Helsinki. This analysis used de‐identified data obtained under credentialed access, with no participant contact, re‐identification, or redistribution of participant data. No study‐specific secondary‐analysis approval, exemption, or waiver is asserted.

## Consent

A waiver of informed consent was granted by the IRB of Beth Israel Deaconess Medical Center for the de‐identified databases analyzed; no participant was contacted and no individual consent was sought for this secondary analysis.

## Conflicts of Interest

The author declares no conflicts of interest.

## Supporting information


**Supporting Information S1:** Supplementary Methods.
**eTable 1:** Excluded Stays Compared With the Analysis Population.
**eTable 2:** Chief‐Complaint Category, Triage Vital Signs, and Missingness by Age Band.
**eTable 3:** Reference Risk, Observed to Expected Ratio by ESI Level, and Age Band by ESI Level Interaction Test.
**eTable 4:** Outcome Components and Discrimination of the ESI Level for Each Component by Age Band.
**eTable 5:** Absolute Performance of the ESI Level at Two Thresholds by Age Band.
**eTable 6:** Case Mix: Stratified and Reweighted Comparisons of the C Statistic, 80 year and Older Versus 18–64 year.
**eFigure 1:** Age Difference in the C Statistic Across the Primary and Additional Analyses.
**eTable 7:** Index Stays Only (First ED Stay of Each Patient).
**eTable 8:** Alternative Outcome Windows: Discrimination and Transportability by Age Band.
**eTable 9:** C Statistic by Anchor‐Year Group.
**eTable 10:** Ratio of Standard Errors With and Without Allowance for Repeat Stays by the Same Patient.
**eTable 11:** Missing Information: Bounds and Scenarios for Stays Without a Recorded ESI Level and for Transfers.
**eTable 12:** Chief‐Complaint Category Against the First‐Listed ED Diagnosis.
**eTable 13:** ED Disposition, Deaths Within 7 and 30 Days of Arrival, and Deaths Not Counted in the Critical Outcome.

**Supporting Information S2:** Reporting Checklists.

## Data Availability

The MIMIC‐IV‐ED data are available from PhysioNet (https://physionet.org/content/mimic‐iv‐ed/2.2/) to credentialed researchers who complete the required training and data‐use agreement; the author is not permitted to redistribute them. The MIMIC‐IV data are available from PhysioNet (https://physionet.org/content/mimiciv/3.1/) to credentialed researchers who complete the required training and data‐use agreement; the author is not permitted to redistribute them. The R analysis code and the dated analysis plan are available from the author on request; the statistical methods, variable definitions, model specifications, and software versions needed to reproduce the analyses are reported in the Methods and the Supporting Information.
