# Supplementary material for: Age Differences in the Discrimination and Level‐Specific Risk of Emergency Severity Index Triage for Critical Outcomes in the Oldest‐Old
Source: Geriatr Gerontol Int. 2026 Nov 8;26(11):e70900. doi: 10.1111/ggi.70900 (PMC13745862; doi:10.1111/ggi.70900)
Supplement: Supplementary file 1 [file GGI-26-0-s001.docx]

**Supporting Information S2**

**Age Differences in the Discrimination and Level-Specific Risk of Emergency Severity Index Triage for Critical Outcomes in the Oldest-Old**

Geriatrics & Gerontology International · 2348259

# Reporting Checklists

Locations are given by section and paragraph (P), counted from the first paragraph under the heading; abstract locations name the field (Aim, Methods, Results, Conclusions).

### STROBE Checklist

| Item | Recommendation | Status and location |
| --- | --- | --- |
| STROBE 1a | Indicate the study’s design with a commonly used term in the title or the abstract. | Met: Abstract, Methods, “Retrospective cohort of 418 025 ED stays of 201 214 adults with an ESI level at one US academic center, 2011-2019 (MIMIC-IV-ED).” |
| STROBE 1b | Provide in the abstract an informative and balanced summary of what was done and what was found. | Met: Abstract, structured summary in four fields: Aim, Methods, Results, and Conclusions. |
| STROBE 2 | Explain the scientific background and rationale for the investigation being reported. | Met: Introduction P1, “yet one Emergency Severity Index (ESI) governs how quickly they are seen… its decision points do not weight for age”; Introduction P2, “None had a younger comparison group… No study has estimated ESI discrimination for death or early intensive care across age bands in a large US cohort”. |
| STROBE 3 | State specific objectives, including any prespecified hypotheses. | Met: Introduction P3, “It compared ESI discrimination and the transportability of level-specific risk for death or intensive care admission within 24 hours between stays at 80 years and older and at 18 to 64 years.”; “It also examined whether under-triage of stays with this critical outcome differed by age after adjustment for triage vital signs and chief complaint.” |
| STROBE 4 | Present key elements of study design early in the paper. | Met: Methods, Study Design and Oversight P1, “This retrospective cohort study used adult ED stays at Beth Israel Deaconess Medical Center, Boston, from 2011 to 2019.”; “Data came from the MIMIC-IV-ED database (version 2.2), linked to MIMIC-IV (version 3.1) for hospital and intensive care unit (ICU) records.” |
| STROBE 5 | Describe the setting, locations, and relevant dates, including periods of recruitment, exposure, follow-up, and data collection. | Met: Methods, Study Design and Oversight P1, “Beth Israel Deaconess Medical Center, Boston… from 2011 to 2019”; Methods, Participants P1, “All ED stays were eligible.” |
| STROBE 6a | Give eligibility criteria, sources, and methods of participant selection.• **Cohort**: Describe methods of follow-up.• **Case-control**: Sources/methods of case ascertainment and control selection; rationale for choices.• **Cross-sectional**: Methods of participant selection. | Met: Methods, Participants P1, “All ED stays were eligible. Stays were excluded, in order, if age at arrival could not be derived or was under 18, or if no ESI level was recorded.”; Methods, Outcomes P1, “or first ICU arrival more than 0 and at most 24 hours after ED arrival” (fixes the outcome-ascertainment window). |
| STROBE 6b | For matched studies, give matching criteria and numbers:• **Cohort**: Number of exposed and unexposed.• **Case-control**: Number of controls per case. | Not addressed: no matching was performed, so no matching-criteria statement was expected or found. |
| STROBE 7 | Clearly define all outcomes, exposures, predictors, potential confounders, and effect modifiers. Give diagnostic criteria, if applicable. | Met: Methods, Outcomes P1 (critical outcome and under-triage defined); Methods, Measurements P1 (ESI level, vital signs, chief complaint, arrival mode, sex defined); Methods, Statistical Analysis P2, “adjusted for the five vital signs (restricted cubic splines, three knots) and chief-complaint category”. |
| STROBE 8* | For each variable of interest, give sources of data and details of methods of assessment (measurement). Describe comparability of assessment methods if more than one group. | Met: Methods, Measurements P1, “The triage nurse assigned the ESI level, from 1 (highest acuity) to 5.”; “values outside plausibility ranges, set before analysis, were treated as missing”; “The free-text chief complaint was mapped by keyword to 16 categories”; eTable 2 (missing counts) and eTable 2, Panel D (plausibility ranges); Supporting Information, eMethods, ‘Plausibility ranges for triage vital signs’ and ‘Chief-complaint categories’ (the keyword-pattern table). |
| STROBE 9 | Describe any efforts to address potential sources of bias. | Met: Methods, Statistical Analysis P2, “with variances cluster-robust on patient”; “Models were unadjusted and adjusted for the five vital signs (restricted cubic splines, three knots) and chief-complaint category”; Results, Case Mix P1, “With stays at 80 and older reweighted to the covariate distribution of younger stays”; Discussion, Limitations P1, “Under-triage odds were adjusted only for triage vital signs and chief complaint. The keyword mapping of chief complaint agreed only moderately with ED diagnoses, and functional status, comorbidity, and goals of care were unmeasured.” Unmeasured confounding is named as a limitation, not quantified or bounded. |
| STROBE 10 | Explain how the study size was arrived at. | Met: Supporting Information, eMethods, ‘Under-triage model’, “No sample size was calculated, the cohort being fixed; the adjusted under-triage model carried 31 parameters against the approximately 2900 under-triage events anticipated at design, more than 90 per parameter.” |
| STROBE 11 | Explain how quantitative variables were handled in the analyses. If applicable, describe which groupings were chosen and why. | Met: Methods, Participants P1, “Age bands were 18 to 64 years (reference), 65 to 74, 75 to 79, 80 to 84, 85 to 89, and 90 and older”; Methods, Statistical Analysis P2, “adjusted for the five vital signs (restricted cubic splines, three knots)”; Supporting Information, eMethods, ‘Under-triage model’, “knots at the 10th, 50th, and 90th percentiles”. |
| STROBE 12a | Describe all statistical methods, including those used to control for confounding. | Met: Methods, Statistical Analysis P1, “Discrimination was the nonparametric C statistic of the ESI level within each band: the probability that a stay with the critical outcome had been assigned a higher-acuity level than a stay without it, with ties counted one half.”; “applying the observed risk at each level at 18 to 64 years (levels 4 and 5 combined) to each older band as its expected risk”; Methods, Statistical Analysis P2, “Under-triage was analyzed among stays with a critical outcome by logistic regression on age band, with variances cluster-robust on patient.”; “Models were unadjusted and adjusted for the five vital signs (restricted cubic splines, three knots)”; Supporting Information, eMethods, ‘Discrimination’, ‘Transportability of level-specific risk’, and ‘Under-triage model’. |
| STROBE 12b | Describe any methods used to examine subgroups and interactions. | Met: Methods, Statistical Analysis P3, “an age band by ESI level interaction”; Supporting Information, eMethods, ‘Additional analyses’, “tested as one cluster-robust Wald statistic on the interaction terms”; Results, Additional Analyses P1, “An age band by ESI level interaction test gave a Wald χ²₂₀ of 1075.6 (P < .001).”; subgroup analyses within arrival-mode and chief-complaint strata, Supporting Information, eMethods, ‘Additional analyses’ (Case mix), “Within each stratum of arrival mode (walk-in, ambulance, other or unknown) and, separately, of chief-complaint category”, and Results, Case Mix P1, “Within arrival-mode strata, the C statistic difference was -0.016 (95% CI, -0.025 to -0.007) among ambulance arrivals”. |
| STROBE 12c | Explain how missing data were addressed. | Met: Methods, Statistical Analysis P2, “Missing vital signs, assumed missing at random given covariates, were imputed by predictive mean matching (20 data sets, 10 iterations)”; Methods, Statistical Analysis P3, “Additional analyses covered index stays, complete cases”; Results, Additional Analyses P1, “In the complete-case analysis (18 606 of 25 407 outcome stays)”; stays without an ESI level, Results, Additional Analyses P2, “When all stays without an ESI level were placed at ESI 1”. |
| STROBE 12d | • **Cohort**: If applicable, explain how loss to follow-up was addressed.• **Case-control**: If applicable, explain how matching of cases and controls was addressed.• **Cross-sectional**: If applicable, describe analytical methods taking account of sampling strategy. | Met: Results, Participants P2, “In the 220 226 stays (52.7%) without a linked admission, the in-hospital death flag was absent and was counted as no event.”; Results, Additional Analyses P2, “Counting all 438 transfers at 80 and older and 1499 at 18 to 64 as having the outcome gave -0.063 (95% CI, -0.071 to -0.056)”; Discussion, Limitations P1, “The composite outcome missed deaths within 7 days after stays without a linked admission: 39 (0.35% of composite events) at 18 to 64 years and 54 (0.85%) at 80 and older (eTable 13)”; “Intensive care admission after transfer could not be observed.”. |
| STROBE 12e | Describe any sensitivity analyses. | Met: Methods, Statistical Analysis P3, “Additional analyses covered index stays, complete cases, an age band by ESI level interaction, alternative outcome windows, fuller adjustment, and anchor-year groups”; “standard-error ratios served as a clustering diagnostic”; “Analyses specified after the primary results were known covered outcome components; absolute performance; discrimination within arrival-mode and chief-complaint strata, pooled by inverse variance, and after inverse-odds reweighting on pre-triage covariates; and bounds for stays without an ESI level and for transfers”; Supporting Information, eMethods, ‘Additional analyses’. |
| STROBE 13a | Report numbers of individuals at each stage of study (e.g. numbers potentially eligible, examined for eligibility, confirmed eligible, included in the study, completing follow-up, and analyzed). | Met: Results, Participants P1, “Of 425 087 ED stays of 205 504 patients, 76 were excluded because age could not be derived and 6986 because no ESI level was recorded (Figure 1), leaving 418 025 stays of 201 214 patients.”; Figure 1 legend, “no stay was excluded for age under 18 years” |
| STROBE 13b | Give reasons for non-participation at each stage. | Met: Results, Participants P1, same sentence as STROBE 13a, giving the specific reason and count for each of the three exclusion steps; Results, Participants P1, “(eTable 1)” for the excluded stays without an ESI level. |
| STROBE 13c | Consider use of a participant flow diagram. | Met: Figure 1, Participant Flow (legend: “with the number of stays remaining after each exclusion”), showing the three exclusion steps and the analysis population (n=418 025 stays; 201 214 patients). |
| STROBE 14a | Give characteristics of study participants (e.g. demographic, clinical, social) and information on exposures and potential confounders. | Met: Table 1 (age, sex, arrival mode, ESI level, admission and outcomes, overall and by age band); eTable 2 (chief complaint and vital signs by band); eTable 1 (excluded stays). |
| STROBE 14b | Indicate number of participants with missing data for each variable of interest. | Met: Results, Participants P2, “In the 220 226 stays (52.7%) without a linked admission, the in-hospital death flag was absent and was counted as no event. After implausible values were set to missing, heart rate was missing in 10 313 stays (2.5%) and in 4660 (18.3%) of stays with a critical outcome (eTable 2).”; eTable 2 (missingness by band and outcome); eTable 2, Panel D (plausibility ranges); eTable 1, Panel B (stays without a recorded ESI level). |
| STROBE 14c | • **Cohort**: Summarize follow-up time (e.g. average and total amount). | Not addressed: fixed-window outcome ascertainment (critical outcome assessed within a strict 24-hour window from Methods Outcomes), not a variable-duration follow-up cohort, so no average or total follow-up-time statement was expected. |
| STROBE 15 | • **Cohort**: Report numbers of outcome events or summary measures over time.• **Case-control**: Report numbers in each exposure category, or summary measures of exposure.• **Cross-sectional**: Report numbers of outcome events or summary measures. | Met: Results, Participants P1, “The critical outcome occurred in 25 407 stays (6.1%); 23 788 of these were ICU admissions within 24 hours. Among 49 731 stays at 80 and older, the critical outcome occurred in 6338 (12.7%), versus 11 209 of 287 140 (3.9%) at 18 to 64 (Table 1).” |
| STROBE 16a | Give unadjusted estimates and, if applicable, confounder-adjusted estimates and their precision (e.g. 95% confidence interval). Make clear which confounders were adjusted for and why they were included. | Met: Results, Under-Triage P1, “The odds ratio for under-triage at 80 and older versus 18 to 64 was 0.77 (95% CI, 0.70-0.85) unadjusted and 0.81 (95% CI, 0.73-0.90) after adjustment for vital signs and chief-complaint category (Table 3).” |
| STROBE 16b | Report category boundaries when continuous variables were categorized. | Met: Methods, Participants P1, “Age bands were 18 to 64 years (reference), 65 to 74, 75 to 79, 80 to 84, 85 to 89, and 90 and older”; Supporting Information, eMethods, ‘Under-triage model’ (spline knot percentiles). |
| STROBE 16c | If relevant, consider translating estimates of relative risk into absolute risk for a meaningful time period. | Met: Results, Under-Triage P1, “Per 100 stays overall in each band, critical-outcome stays at ESI 3 to 5 numbered 1.42 (95% CI, 1.32-1.53) at 80 and older and 0.55 (95% CI, 0.52-0.58) at 18 to 64.”; Results, Participants P1 (absolute rates, 12.7% versus 3.9%); Table 3, Panel B (absolute performance by age band). |
| STROBE 17 | Report other analyses done (e.g. analyses of subgroups and interactions, and sensitivity analyses). | Met: Results, Additional Analyses P1, “In the complete-case analysis (18 606 of 25 407 outcome stays), the adjusted odds ratio at 80 and older was 0.78 (95% CI, 0.70-0.87)”; “With arrival mode, sex, and anchor-year group added, it was 0.77 (95% CI, 0.69-0.85)” and the index-stays, interaction, alternative-window, and anchor-year-group results (eTables 3 and 7 to 9) and clustering diagnostics (eTable 10); Results, Additional Analyses P2, bounds for stays without an ESI level and for transfers (eTable 11) and complaint-mapping agreement (eTable 12); Results, Discrimination and Level-Specific Risk P2, outcome components (eTable 4), and P3, absolute performance (Table 3); Results, Case Mix P1, arrival-mode and chief-complaint strata and reweighting (eTable 6). |
| STROBE 18 | Summarize key results with reference to study objectives. | Met: Discussion P1, “In this single-center cohort, ESI levels ranked critical-outcome risk less well at 80 years and older than at 18 to 64, and the shortfall was present in every older band, from -0.044 at 65 to 74 to -0.069 at both 85 to 89 and 90 and older.” |
| STROBE 19 | Discuss limitations of the study, taking into account sources of potential bias or imprecision. Discuss both direction and magnitude of any potential bias. | Met: Discussion, Limitations P1 (the subsection beginning “The composite outcome missed deaths within 7 days”), covering outcome ascertainment after stays without a linked admission and after transfer, the bounds for stays without an ESI level, the single center with time-shifted dates, the limited adjustment set and moderate complaint-mapping agreement, and the absence of chart review; imprecision is not discussed as a limitation in itself (intervals are reported throughout Results). |
| STROBE 20 | Give a cautious overall interpretation of results considering objectives, limitations, multiplicity of analyses, results from similar studies, and other relevant evidence. | Met: Discussion, Possible Explanations P1, “This design cannot establish why the levels rank risk less well with age; the explanations below are inferences.”; Discussion, Implications P1, “The finding does not show that an age-specific threshold or a geriatric flag would improve outcomes.”; “It identifies where level-specific risk differs most by age.”; “A retrospective single-center analysis does not by itself support changing practice”; Discussion, Implications P1 (last sentence), “whether an age-specific rule could help needs prospective study”; Methods, Statistical Analysis P3, “Confidence intervals for secondary and additional analyses were not adjusted for multiplicity and do not support definitive inference.” |
| STROBE 21 | Discuss the generalizability (external validity) of the study results. | Met: Discussion, Limitations P1, “The cohort is one academic center with time-shifted dates, so the size of the gap elsewhere is untested.” names the single-center limit and states that the size of the gap in other settings is untested; Discussion, Earlier Studies P1 compares the findings with Finnish, Japanese, and Korean studies. |
| STROBE 22 | Give the source of funding and the role of the funders for the present study and, if applicable, for the original study on which the present article is based. | Met: Methods, Study Design and Oversight P1, “The analysis plan was dated before analysis but not registered.”; Funding Statement, “This research received no specific grant from any funding agency in the public, commercial, or not-for-profit sectors.” |

### RECORD Checklist

| Item | Recommendation | Status and location |
| --- | --- | --- |
| RECORD 1 | (a) Indicate study design with commonly used term in title/abstract.(b) Provide informative and balanced summary in abstract. | Partial; base met, same as STROBE 1a/1b (Abstract, Methods, and the four-field structured abstract). RECORD 1.1: Met, Abstract, Methods, “(MIMIC-IV-ED)”; Methods, Study Design and Oversight P1, “MIMIC-IV-ED database (version 2.2)”. RECORD 1.2: Met, Abstract, Methods, “at one US academic center, 2011-2019 (MIMIC-IV-ED)” (region and timeframe). RECORD 1.3: Not addressed in the abstract, which does not state the linkage; stated at Introduction P3, “linked to hospital and intensive care records”, and Methods, Study Design and Oversight P1, “linked to MIMIC-IV (version 3.1)”. |
| RECORD 2 | Explain scientific background and rationale for investigation. | Met: same as STROBE 2 (no RECORD extension applies). |
| RECORD 3 | State specific objectives, including prespecified hypotheses. | Met: same as STROBE 3 (no RECORD extension applies). |
| RECORD 4 | Present key elements of study design early in paper. | Met: same as STROBE 4 (no RECORD extension applies). |
| RECORD 5 | Describe setting, locations, relevant dates (recruitment, exposure, follow-up, collection). | Met: same as STROBE 5 (no RECORD extension applies). |
| RECORD 6 | (a) Eligibility criteria, sources and methods of selection, follow-up methods.(b) Matching criteria and numbers if applicable. | Base met, same as STROBE 6a. RECORD 6.1: met, Supporting Information, eMethods, ‘Chief-complaint categories’ (the full keyword-pattern table by category) and ‘Plausibility ranges for triage vital signs’ (ranges in eTable 2, Panel D). RECORD 6.2: none found, no validation-study citation for any code or algorithm appears anywhere. RECORD 6.3: partial, Figure 1 diagrams the three exclusion steps only; the ED-to-hospital-to-ICU linkage step is disclosed only in prose at Results, Participants P2, “220 226 stays (52.7%) without a linked admission”. |
| RECORD 7 | Clearly define all outcomes, exposures, predictors, potential confounders, effect modifiers; give diagnostic criteria. | Base met, same as STROBE 7. RECORD 7.1: met for the vital signs and the chief complaint, eTable 2, Panel D giving the complete plausibility ranges and Supporting Information, eMethods giving the complete keyword algorithm by category; partial for the critical outcome, whose MIMIC-IV field-level derivation is not printed, only its plain-English definition at Methods, Outcomes P1. |
| RECORD 8 | For each variable, give sources of data and details of assessment methods. | Met: same as STROBE 8* (no RECORD extension applies). |
| RECORD 9 | Describe any efforts to address potential sources of bias. | Met: same as STROBE 9 (Discussion, Limitations P1 names unmeasured confounding without quantifying it); no RECORD extension applies. |
| RECORD 10 | Explain how study size was arrived at. | Met: same as STROBE 10 (no RECORD extension applies). |
| RECORD 11 | Explain how quantitative variables were handled in analyses (groupings, cutoffs). | Met: same as STROBE 11 (no RECORD extension applies). |
| RECORD 12 | (a) Describe all statistical methods.(b) Methods for subgroups/interactions.(c) Missing data handling.(d) Cohort/case-control/cross-sectional specifics.(e) Sensitivity analyses. | Base met, same as STROBE 12a-e. RECORD 12.1: partial, Methods, Statistical Analysis P2, “with variances cluster-robust on patient” addresses clustering, and the multiple-imputation sentence addresses missing recording; the chief-complaint mapping is checked against the first-listed ED diagnosis, Results, Additional Analyses P2, “the chief-complaint category agreed with the first-listed ED diagnosis in 49.1% to 67.5% of trauma, chest pain, and respiratory complaints at 80 and older” (eTable 12); no method addressing misclassification of the ESI level itself is described. RECORD 12.2: none found, the 52.7% unlinked rate is reported (Results, Participants P2) and the deaths the composite could miss after unlinked stays are counted (eTable 13), but no method for assessing linkage quality, error rate, or false-match rate is described anywhere. |
| RECORD 13 | (a) Report numbers of individuals at each stage.(b) Reasons for non-participation.(c) Use participant flow diagram. | Base met, same as STROBE 13a-c. RECORD 13.1: partial, Figure 1 diagrams the three exclusion steps and the final analysis population but not the linkage process itself, same caveat as RECORD 6.3. |
| RECORD 14 | (a) Characteristics of participants (demographic, clinical, social).(b) Number of participants with missing data.(c) Cohort follow-up time summary. | Met as to 14a-14b (same as STROBE 14a-b); Not addressed as to 14c (same as STROBE 14c); no RECORD extension applies. |
| RECORD 15 | Report numbers of outcome events or summary measures over time. | Met: same as STROBE 15 (no RECORD extension applies). |
| RECORD 16 | (a) Unadjusted estimates and confounder-adjusted estimates with 95% CIs.(b) Report category boundaries.(c) Relative and absolute risk estimates. | Met: same as STROBE 16a-c (no RECORD extension applies). |
| RECORD 17 | Report other analyses done (subgroups, interactions, sensitivity analyses). | Met: same as STROBE 17 (no RECORD extension applies). |
| RECORD 18 | Summarize key results with reference to study objectives. | Met: same as STROBE 18 (no RECORD extension applies). |
| RECORD 19 | Discuss limitations taking into account sources of bias or imprecision. | Base met, same as STROBE 19. RECORD 19.1: met, Discussion, Limitations P1, “functional status, comorbidity, and goals of care were unmeasured” (residual confounding); “because the completeness of death recording after such stays is unknown, the size and direction of any resulting bias are unknown” (linkage and missing outcome data); “The keyword mapping of chief complaint agreed only moderately with ED diagnoses” (misclassification of the chief-complaint mapping); “so instrument failure and assignment failure cannot be separated” (ESI assignment, named without a separate misclassification statement). |
| RECORD 20 | Give cautious overall interpretation of results considering objectives, limitations, multiple analyses, similar studies. | Met: same as STROBE 20 (no RECORD extension applies). |
| RECORD 21 | Discuss external validity/generalizability of study results. | Met: same as STROBE 21 (no RECORD extension applies). |
| RECORD 22 | Give source of funding and role of funders for present study. | Met: same as STROBE 22 (no RECORD extension applies). |
| RECORD 22.1 | Provide statement on data accessibility, code availability, metadata sharing, and access permissions for the databases used. | Met: Data Availability Statement, “The MIMIC-IV-ED data are available from PhysioNet (https://physionet.org/content/mimic-iv-ed/2.2/) to credentialed researchers who complete the required training and data-use agreement” … “The R analysis code and the dated analysis plan are available from the author on request”. |

### TRIPOD+AI Checklist

| Item | Recommendation | Status and location |
| --- | --- | --- |
| TRIPOD+AI 1 | Identify the study as developing or evaluating the performance of a multivariable prediction model, the target population, and the outcome to be predicted | Met: Title, “Age Differences in the Discrimination and Level-Specific Risk of Emergency Severity Index Triage for Critical Outcomes in the Oldest-Old” (names the instrument, the population, and the outcome); Methods, Study Design and Oversight P1, “and developed no model.” (evaluation of an existing instrument). |
| TRIPOD+AI 2 | See TRIPOD+AI for Abstracts checklist | Met: Abstract, four-field structured summary (Aim, Methods, Results, Conclusions); see the TRIPOD+AI for Abstracts checklist below. |
| TRIPOD+AI 3a | Explain the healthcare context (including whether diagnostic or prognostic) and rationale for developing or evaluating the prediction model, including references to existing models | Partial: Introduction P1, “The ESI was validated in patients of all ages, but older patients often present with blunted vital signs and nonspecific complaints” (healthcare context); Introduction P2, “In single-center studies of patients aged 65 and older, ESI level 1 missed most of those receiving an immediate life-saving intervention” (rationale and earlier evaluations of the existing instrument, references 1 to 6); the text does not say in words whether the instrument is used diagnostically or prognostically. |
| TRIPOD+AI 3b | Describe the target population and the intended purpose of the prediction model in the context of the care pathway, including its intended users (eg, healthcare professionals, patients, public) | Met: Introduction P1, “yet one Emergency Severity Index (ESI) governs how quickly they are seen” (place in the care pathway); “An age-specific threshold or a geriatric flag at triage is worth testing only if the ESI levels rank and quantify risk less well in the oldest-old.” (purpose); Methods, Measurements P1, “The triage nurse assigned the ESI level, from 1 (highest acuity) to 5.” (user); Methods, Participants P1, “the oldest-old were 80 and older” (target population). |
| TRIPOD+AI 3c | Describe any known health inequalities between sociodemographic groups | Partial: Introduction P1, “have a higher risk of death or early intensive care than younger adults” (age as the sociodemographic axis); no other sociodemographic inequality (sex, race and ethnicity, language, insurance) is described. |
| TRIPOD+AI 4 | Specify the study objectives, including whether the study describes the development or validation of a prediction model (or both) | Met: Introduction P3, “It compared ESI discrimination and the transportability of level-specific risk for death or intensive care admission within 24 hours between stays at 80 years and older and at 18 to 64 years.”; Methods, Study Design and Oversight P1, “It evaluated an existing instrument, the ESI,” and “developed no model.” (evaluation, not development). |
| TRIPOD+AI 5a | Describe the sources of data separately for the development and evaluation datasets (eg, randomised trial, cohort, routine care or registry data), the rationale for using these data, and representativeness of the data | Partial: Methods, Study Design and Oversight P1, “Data came from the MIMIC-IV-ED database (version 2.2),” (routine-care source; one evaluation dataset, no development dataset); Discussion, Limitations P1, “The cohort is one academic center with time-shifted dates, so the size of the gap elsewhere is untested.” (representativeness); no sentence states why MIMIC-IV-ED was chosen. |
| TRIPOD+AI 5b | Specify the dates of the collected participant data, including start and end of participant accrual; and, if applicable, end of follow-up | Met: Methods, Study Design and Oversight P1, “from 2011 to 2019.” (accrual period); Methods, Outcomes P1, “at most 24 hours after ED arrival” (follow-up for the outcome); Methods, Statistical Analysis P3, “anchor-year groups (patient-level de-identified calendar windows)” (dates in the source are shifted). |
| TRIPOD+AI 6a | Specify key elements of the study setting (eg, primary care, secondary care, general population) including the number and location of centres | Met: Methods, Study Design and Oversight P1, “adult ED stays at Beth Israel Deaconess Medical Center, Boston” (secondary-care emergency department, one centre, location); Abstract, Methods, “at one US academic center”. |
| TRIPOD+AI 6b | Describe the eligibility criteria for study participants | Met: Methods, Participants P1, “All ED stays were eligible. Stays were excluded, in order, if age at arrival could not be derived or was under 18, or if no ESI level was recorded.” |
| TRIPOD+AI 6c | Give details of any treatments received, and how they were handled during model development or evaluation, if relevant | Not applicable: treatments were not part of any estimand and none is reported; Discussion, Limitations P1 states “functional status, comorbidity, and goals of care were unmeasured.” |
| TRIPOD+AI 7 | Describe any data pre-processing and quality checking, including whether this was similar across relevant sociodemographic groups | Met: Methods, Measurements P1, “values outside plausibility ranges, set before analysis, were treated as missing (eTable 2)” and “The free-text chief complaint was mapped by keyword to 16 categories (eMethods).”; Methods, Statistical Analysis P3, “Complaint-mapping agreement was described (eMethods).”; Results, Participants P1, “Stays without an ESI level made up 2.1% of stays at 80 years and older and 1.5% at 18 to 64 years.” (quality checks by age band; missingness by band in eTable 2). |
| TRIPOD+AI 8a | Clearly define the outcome that is being predicted and the time horizon, including how and when assessed, the rationale for choosing this outcome, and whether the method of outcome assessment is consistent across sociodemographic groups | Met: Methods, Outcomes P1, “The critical outcome was death in the ED or during the linked hospitalization, or first ICU arrival more than 0 and at most 24 hours after ED arrival.” (definition and time horizon); Introduction P1, “have a higher risk of death or early intensive care than younger adults” (rationale); Discussion, Possible Explanations P2, “Fourth, intensive care admission depends on goals of care and comorbidity, which triage does not capture.” (consistency of assessment across age groups is discussed). |
| TRIPOD+AI 8b | If outcome assessment requires subjective interpretation, describe the qualifications and demographic characteristics of the outcome assessors | Not applicable: the outcome was derived by rule from recorded death and intensive care fields (Methods, Outcomes P1, “first ICU arrival more than 0 and at most 24 hours after ED arrival”); no assessor interpretation was involved. |
| TRIPOD+AI 8c | Report any actions to blind assessment of the outcome to be predicted | Not addressed: no statement on blinding of outcome assessment appears; the outcome was taken from recorded database fields. |
| TRIPOD+AI 9a | Describe the choice of initial predictors (eg, literature, previous models, all available predictors) and any pre-selection of predictors before model building | Not applicable: development-only item; the study evaluated an existing instrument and developed no model. |
| TRIPOD+AI 9b | Clearly define all predictors, including how and when they were measured (and any actions to blind assessment of predictors for the outcome and other predictors) | Met: Methods, Measurements P1, “The triage nurse assigned the ESI level, from 1 (highest acuity) to 5.” and “Triage vital signs were heart rate, systolic blood pressure, respiratory rate, oxygen saturation, and temperature”; “Arrival mode and sex were recorded”; the ESI level is assigned at triage, before the outcome; no blinding action is reported. |
| TRIPOD+AI 9c | If predictor measurement requires subjective interpretation, describe the qualifications and demographic characteristics of the predictor assessors | Partial: Methods, Measurements P1, “The triage nurse assigned the ESI level, from 1 (highest acuity) to 5.” names the assessor of the ESI level; the nurses’ qualifications and demographic characteristics are not described. |
| TRIPOD+AI 10 | Explain how the study size was arrived at (separately for development and evaluation), and justify that the study size was sufficient to answer the research question. Include details of any sample size calculation | Met: Supporting Information, eMethods, Under-triage model, “No sample size was calculated, the cohort being fixed; the adjusted under-triage model carried 31 parameters against the approximately 2900 under-triage events anticipated at design, more than 90 per parameter.” (one evaluation dataset). |
| TRIPOD+AI 11 | Describe how missing data were handled. Provide reasons for omitting any data | Met: Methods, Statistical Analysis P2, “Missing vital signs, assumed missing at random given covariates, were imputed by predictive mean matching (20 data sets, 10 iterations)”; Results, Participants P1, “76 were excluded because age could not be derived and 6986 because no ESI level was recorded” (reasons for omitting data); Results, Participants P2, “In the 220 226 stays (52.7%) without a linked admission, the in-hospital death flag was absent and was counted as no event.” |
| TRIPOD+AI 12a | Describe how the data were used (eg, for development and evaluation of model performance) in the analysis, including whether the data were partitioned, considering any sample size requirements | Not applicable: development-only item; the study evaluated an existing instrument and developed no model. |
| TRIPOD+AI 12b | Depending on the type of model, describe how predictors were handled in the analyses (functional form, rescaling, transformation, or any standardisation) | Not applicable: development-only item; the study evaluated an existing instrument and developed no model. |
| TRIPOD+AI 12c | Specify the type of model, rationale†, all model building steps, including any hyperparameter tuning, and method for internal validation | Not applicable: development-only item; the study evaluated an existing instrument and developed no model. |
| TRIPOD+AI 12d | Describe if and how any heterogeneity in estimates of model parameter values and model performance was handled and quantified across clusters (eg, hospitals, countries). See TRIPOD-Cluster for additional considerations | Not applicable: one centre, so no heterogeneity across hospitals or countries exists to quantify; repeat stays by the same patient were handled by cluster-robust variances (Methods, Statistical Analysis P2, “with variances cluster-robust on patient”). |
| TRIPOD+AI 12e | Specify all measures and plots used (and their rationale) to evaluate model performance (eg, discrimination, calibration, clinical utility) and, if relevant, to compare multiple models | Met: Methods, Outcomes P1, “The primary estimands were the discrimination (C statistic) of the ESI level for the critical outcome and the transportability of the risk carried by each level”; Methods, Statistical Analysis P1, “Because the ESI assigns levels, not probabilities, transportability was assessed by applying the observed risk at each level at 18 to 64 years” (rationale) and “Agreement was summarized by a transport intercept (logistic regression with the logit of the expected risk as offset), a transport slope (regression on that logit), and observed to expected ratios”; Discussion, Ranking and Absolute Risk P2, “Ranking and absolute risk are separate properties of any risk instrument”; Figure 2 legend (Figure Legends, “Observed risk of the critical outcome by Emergency Severity Index (ESI) level and age band.”); no clinical-utility measure is reported and no models are compared. |
| TRIPOD+AI 12f | Describe any model updating (eg, recalibration) arising from the model evaluation, either overall or for particular sociodemographic groups or settings | Not applicable: the ESI was used exactly as recorded; no recalibration or other updating was performed (Methods, Study Design and Oversight P1, “and developed no model.”). |
| TRIPOD+AI 12g | For model evaluation, describe how the model predictions were calculated (eg, formula, code, object, application programming interface) | Met: Methods, Measurements P1, “The triage nurse assigned the ESI level, from 1 (highest acuity) to 5.”; Methods, Statistical Analysis P1, “Because the ESI assigns levels, not probabilities, transportability was assessed by applying the observed risk at each level at 18 to 64 years” (the expected risk for each level is the observed risk at 18 to 64 years; Supporting Information, eMethods, Transportability of level-specific risk, “The reference proportions were treated as fixed.”). |
| TRIPOD+AI 13 | If class imbalance methods were used, state why and how this was done, and any subsequent methods to recalibrate the model or the model predictions | Not applicable: no class imbalance method was used. |
| TRIPOD+AI 14 | Describe any approaches that were used to address model fairness and their rationale | Partial: the study is itself a comparison of instrument performance across age bands (Methods, Outcomes P1, “each estimated by band and contrasted between 80 years and older and 18 to 64 years”); no approach to address fairness was applied, and Discussion, Implications P1 states “The finding does not show that an age-specific threshold or a geriatric flag would improve outcomes.” |
| TRIPOD+AI 15 | Specify the output of the prediction model (eg, probabilities, classification). Provide details and rationale for any classification and how the thresholds were identified | Not applicable: development-only item; the study evaluated an existing instrument and developed no model. |
| TRIPOD+AI 16 | Identify any differences between the development and evaluation data in healthcare setting, eligibility criteria, outcome, and predictors | Not addressed: there is no development dataset in this study, and the setting in which the ESI was developed is not compared with this cohort (Introduction P1, “The ESI was validated in patients of all ages” is the only statement); the age bands share one setting, one eligibility rule, one outcome and one set of predictors (Methods, Participants P1). |
| TRIPOD+AI 17 | Name the institutional research board or ethics committee that approved the study and describe the participant informed consent or the ethics committee waiver of informed consent | Met: Ethics Approval Statement P1, “MIMIC-IV and MIMIC-IV-ED were each approved by the Institutional Review Board (IRB) of Beth Israel Deaconess Medical Center” and “which granted a waiver of informed consent”; Patient Consent Statement P1, “A waiver of informed consent was granted by the IRB of Beth Israel Deaconess Medical Center for the de-identified databases analyzed”; Ethics Approval Statement P1, “No study-specific secondary-analysis approval, exemption, or waiver is asserted.” |
| TRIPOD+AI 18a | Give the source of funding and the role of the funders for the present study | Met: Funding Statement P1, “This research received no specific grant from any funding agency in the public, commercial, or not-for-profit sectors.” |
| TRIPOD+AI 18b | Declare any conflicts of interest and financial disclosures for all authors | Met: Disclosure Statement P1, “The author declares no conflict of interest.” |
| TRIPOD+AI 18c | Indicate where the study protocol can be accessed or state that a protocol was not prepared | Met: Methods, Study Design and Oversight P1, “The analysis plan was dated before analysis but not registered.”; Data Availability Statement P1, “The R analysis code and the dated analysis plan are available from the author on request” |
| TRIPOD+AI 18d | Provide registration information for the study, including register name and registration number, or state that the study was not registered | Met: Clinical Trial Registration P1, “This study is an observational secondary analysis of existing de-identified data and was not registered.” |
| TRIPOD+AI 18e | Provide details of the availability of the study data | Met: Data Availability Statement P1, “The MIMIC-IV-ED data are available from PhysioNet” and “to credentialed researchers who complete the required training and data-use agreement; the author is not permitted to redistribute them.” |
| TRIPOD+AI 18f | Provide details of the availability of the analytical code | Met: Data Availability Statement P1, “The R analysis code and the dated analysis plan are available from the author on request” |
| TRIPOD+AI 19 | Provide details of any patient and public involvement during the design, conduct, reporting, interpretation, or dissemination of the study or state no involvement | Not addressed: no statement on patient and public involvement, or on its absence, appears in the manuscript. |
| TRIPOD+AI 20a | Describe the flow of participants through the study, including the number of participants with and without the outcome and, if applicable, a summary of the follow-up time. A diagram may be helpful | Met: Results, Participants P1, “Of 425 087 ED stays of 205 504 patients, 76 were excluded because age could not be derived and 6986 because no ESI level was recorded (Figure 1), leaving 418 025 stays of 201 214 patients.” and “The critical outcome occurred in 25 407 stays (6.1%)”; Figure 1 legend (Figure Legends, “Participant flow.”); follow-up is the fixed 24-hour outcome window (Methods, Outcomes P1). |
| TRIPOD+AI 20b | Report the characteristics overall and, where applicable, for each data source or setting, including the key dates, key predictors (including demographics), treatments received, sample size, number of outcome events, follow-up time, and amount of missing data. A table may be helpful. Report any differences across key demographic groups | Met: Table 1 (age, sex, arrival mode, ESI level, admission and critical outcome, overall and by age band); Results, Participants P1, “Among 49 731 stays at 80 and older, the critical outcome occurred in 6338 (12.7%), versus 11 209 of 287 140 (3.9%) at 18 to 64 (Table 1).”; Results, Participants P2, “heart rate was missing in 10 313 stays (2.5%) and in 4660 (18.3%) of stays with a critical outcome (eTable 2)”; one source and setting; treatments are not reported (see TRIPOD+AI 6c). |
| TRIPOD+AI 20c | For model evaluation, show a comparison with the development data of the distribution of important predictors (demographics, predictors, and outcome) | Not addressed: no development dataset exists to compare with (see TRIPOD+AI 16); the age bands are compared in Table 1 and eTable 2. |
| TRIPOD+AI 21 | Specify the number of participants and outcome events in each analysis (eg, for model development, hyperparameter tuning, model evaluation) | Met: Table 2, column “Critical outcomes, No.” for every band; Results, Participants P1, “Among 49 731 stays at 80 and older, the critical outcome occurred in 6338 (12.7%), versus 11 209 of 287 140 (3.9%) at 18 to 64 (Table 1).”; Results, Additional Analyses P1, “In the complete-case analysis (18 606 of 25 407 outcome stays)” and “In index stays (201 214)”; Results, Case Mix P1, “(effective sample size 10 389)”. |
| TRIPOD+AI 22 | Provide details of the full prediction model (eg, formula, code, object, application programming interface) to allow predictions in new individuals and to enable third party evaluation and implementation, including any restrictions to access or reuse (eg, freely available, proprietary) | Not applicable: development-only item; the study evaluated an existing instrument and developed no model. |
| TRIPOD+AI 23a | Report model performance estimates with confidence intervals, including for any key subgroups (eg, sociodemographic). Consider plots to aid presentation | Met: Results, Discrimination and Level-Specific Risk P1, “The C statistic of the ESI level was 0.818 (95% CI, 0.814-0.822) at 18 to 64 years and 0.754 (95% CI, 0.748-0.759) at 80 years and older (difference, -0.065; 95% CI, -0.072 to -0.058).” and “the transport intercept at 80 and older was 0.85 (95% CI, 0.82-0.88) and the slope 0.88 (95% CI, 0.85-0.91)”; Table 2 and Table 3 give every age band with 95% confidence intervals; Figure 2 plots observed risk by ESI level and age band. |
| TRIPOD+AI 23b | If examined, report results of any heterogeneity in model performance across clusters. See TRIPOD-Cluster for additional details | Not applicable: one centre, so no clusters of hospitals exist; the only grouping examined is the anchor-year group (Results, Additional Analyses P1, “Across anchor-year groups, the C statistic rose from 0.795 to 0.834 at 18 to 64 and ranged from 0.744 to 0.755 at 80 and older (eTable 9).”). |
| TRIPOD+AI 24 | Report the results from any model updating, including the updated model and subsequent performance | Not applicable: no model updating was performed (see TRIPOD+AI 12f). |
| TRIPOD+AI 25 | Give an overall interpretation of the main results, including issues of fairness in the context of the objectives and previous studies | Met: Discussion P1, “In this single-center cohort, ESI levels ranked critical-outcome risk less well at 80 years and older than at 18 to 64” and “Levels 1 to 3 carried more risk at 80 and older than at 18 to 64.”; Discussion, Earlier Studies P1, “Kemp and colleagues, in a Finnish cohort over one month, found no significant difference in ESI accuracy between patients under and over 65 for any outcome.” (previous studies); Discussion, Implications P1, “The finding does not show that an age-specific threshold or a geriatric flag would improve outcomes.” (age is the only sociodemographic dimension examined). |
| TRIPOD+AI 26 | Discuss any limitations of the study (such as a non-representative sample, sample size, overfitting, missing data) and their effects on any biases, statistical uncertainty, and generalisability | Met: Discussion, Limitations P1, “The cohort is one academic center with time-shifted dates, so the size of the gap elsewhere is untested.” (generalisability); “Because the completeness of death recording after such stays is unknown, the size and direction of any resulting bias are unknown.” (missing data); “Under-triage odds were adjusted only for triage vital signs and chief complaint” (residual confounding); Methods, Statistical Analysis P3, “Confidence intervals for secondary and additional analyses were not adjusted for multiplicity and do not support definitive inference.” |
| TRIPOD+AI 27a | Describe how poor quality or unavailable input data (eg, predictor values) should be assessed and handled when implementing the prediction model | Not applicable: development-only item; the study evaluated an existing instrument and developed no model. |
| TRIPOD+AI 27b | Specify whether users will be required to interact in the handling of the input data or use of the model, and what level of expertise is required of users | Not applicable: development-only item; the study evaluated an existing instrument and developed no model. |
| TRIPOD+AI 27c | Discuss any next steps for future research, with a specific view to applicability and generalisability of the model | Partial: Discussion, Implications P1, “A retrospective single-center analysis does not by itself support changing practice; whether an age-specific rule could help needs prospective study.” and Abstract, Conclusions, “Age-specific triage rules need prospective study.” name prospective study as the next step; no sentence says what that study would test, and generalisability is addressed only by “the size of the gap elsewhere is untested.” (Discussion, Limitations P1). |

### TRIPOD+AI for Abstracts Checklist

| Item | Recommendation | Status and location |
| --- | --- | --- |
| TRIPOD+AI-Abstracts 1 | Identify the study as developing or evaluating the performance of a multivariable prediction model, the target population, and the outcome to be predicted | Met: same Title as TRIPOD+AI item 1. |
| TRIPOD+AI-Abstracts 2 | Provide a brief explanation of the healthcare context and rationale for developing or evaluating the performance of all models | Partial: the abstract has no separate background sentence; Abstract, Aim, “To compare Emergency Severity Index (ESI) discrimination, level-specific risk, and under-triage for a critical outcome between emergency department (ED) stays aged 80 years and older and 18 to 64 years.” gives the context only through the aim. |
| TRIPOD+AI-Abstracts 3 | Specify the study objectives, including whether the study describes model development, evaluation, or both | Met: Abstract, Aim, “To compare Emergency Severity Index (ESI) discrimination, level-specific risk, and under-triage for a critical outcome between emergency department (ED) stays aged 80 years and older and 18 to 64 years.” |
| TRIPOD+AI-Abstracts 4 | Describe the sources of data | Met: Abstract, Methods, “at one US academic center, 2011-2019 (MIMIC-IV-ED)” (names the database and dates); “Retrospective cohort of 418 025 ED stays” (states the design). |
| TRIPOD+AI-Abstracts 5 | Describe the eligibility criteria and setting where the data were collected | Met: Abstract, Methods, “418 025 ED stays of 201 214 adults with an ESI level at one US academic center”. |
| TRIPOD+AI-Abstracts 6 | Specify the outcome to be predicted by the model, including time horizon of predictions in case of prognostic models | Met: Abstract, Methods, “The critical outcome was death in the ED or hospital, or intensive care admission within 24 hours.” |
| TRIPOD+AI-Abstracts 7 | Specify the type of model, a summary of the model-building steps, and the method for internal validation | Not addressed: no candidate-predictor list is reported for this non-model evaluation of a single rule-based instrument; the abstract names only the instrument under evaluation (Abstract, Aim, “Emergency Severity Index (ESI) discrimination”), which is not a predictor list and does not satisfy the item. |
| TRIPOD+AI-Abstracts 8 | Specify the measures used to assess model performance (eg, discrimination, calibration, clinical utility) | Met: Abstract, Methods, “Measures were the ESI C statistic, transportability of each level’s risk from 18 to 64 years, under-triage odds (ESI 3 to 5 in an outcome stay), and differences after case-mix stratification and reweighting.”; the covariates of the adjusted under-triage model are not stated in the abstract. |
| TRIPOD+AI-Abstracts 9 | Report the number of participants and outcome events | Partial: sample size is given, Abstract, Methods, “418 025 ED stays of 201 214 adults with an ESI level”; the critical-outcome event count or rate (6.1% overall; 12.7% at 80 and older, 3.9% at 18 to 64) does not appear in the abstract, which gives only under-triage proportions, Abstract, Results, “Under-triage occurred in 11.1% of outcome stays at 80 and older and 14.0% at 18 to 64”. |
| TRIPOD+AI-Abstracts 10 | Summarise the predictors in the final model | Partial: Abstract, Results, “The C statistic was 0.818 at 18 to 64 and 0.754 at 80 and older (difference, -0.065; 95% CI, -0.072 to -0.058)” and “adjusted odds ratio, 0.81; 95% CI, 0.73-0.90” carry 95% CIs; the band C statistics, “observed risk was 1.96 times that carried by the same levels at 18 to 64”, and the specificity “48.8% versus 69.0%” are given without intervals, and the transport intercept and slope are not in the abstract. |
| TRIPOD+AI-Abstracts 11 | Report model performance estimates (with confidence intervals) | Met: Abstract, Conclusions, “At 80 years and older, ESI levels ranked critical-outcome risk less well, a difference smaller within arrival-mode strata, and levels 1 to 3 carried more risk than in younger adults. Age-specific triage rules need prospective study.” |
| TRIPOD+AI-Abstracts 12 | Give an overall interpretation of the main results | Not addressed: the composed abstract carries no funding, registration, or repository sentence; no Trial Registration field appears in the structured abstract as composed. |
