# Supplementary material for: Age Differences in the Discrimination and Level‐Specific Risk of Emergency Severity Index Triage for Critical Outcomes in the Oldest‐Old
Source: Geriatr Gerontol Int. 2026 Nov 8;26(11):e70900. doi: 10.1111/ggi.70900 (PMC13745862; doi:10.1111/ggi.70900)
Supplement: Supplementary file 2 [file GGI-26-0-s002.docx]

**Supporting Information S1**

**Age Differences in the Discrimination and Level-Specific Risk of Emergency Severity Index Triage for Critical Outcomes in the Oldest-Old**

Geriatrics & Gerontology International · 2348259

**Contents**

**eTable 1**	Excluded Stays Compared With the Analysis Population

**eTable 2**	Chief-Complaint Category, Triage Vital Signs, and Missingness by Age Band

**eTable 3**	Reference Risk, Observed to Expected Ratio by ESI Level, and Age Band by ESI Level Interaction Test

**eTable 4**	Outcome Components and Discrimination of the ESI Level for Each Component by Age Band

**eTable 5**	Absolute Performance of the ESI Level at Two Thresholds by Age Band

**eTable 6**	Case Mix: Stratified and Reweighted Comparisons of the C Statistic, 80 y and Older Versus 18 to 64 y

**eFigure 1**	Age Difference in the C Statistic Across the Primary and Additional Analyses

**eTable 7**	Index Stays Only (First ED Stay of Each Patient)

**eTable 8**	Alternative Outcome Windows: Discrimination and Transportability by Age Band

**eTable 9**	C Statistic by Anchor-Year Group

**eTable 10**	Ratio of Standard Errors With and Without Allowance for Repeat Stays by the Same Patient

**eTable 11**	Missing Information: Bounds and Scenarios for Stays Without a Recorded ESI Level and for Transfers

**eTable 12**	Chief-Complaint Category Against the First-Listed ED Diagnosis

**eTable 13**	ED Disposition, Deaths Within 7 and 30 Days of Arrival, and Deaths Not Counted in the Critical Outcome

## eMethods

### Data definitions

#### Plausibility ranges for triage vital signs

Values outside the plausible ranges in eTable 2, Panel D were set to missing before analysis.

#### Chief-complaint categories

The upper-cased free-text chief complaint was assigned to the first category, in the order listed, whose keyword patterns it matched; a complaint matching none, or blank or redacted, was Other or not recorded.

| Category | Keyword patterns |
| --- | --- |
| Trauma or injury | FALL, MVC, MVA, INJUR, LACERAT, FRACTURE, ASSAULT, TRAUMA, WOUND, BURN, BIKE, PEDESTRIAN, DISLOCAT, AMPUTAT, GSW, STAB, CRUSH, SCOOTER, MOTORCYCLE |
| Psychiatric or substance | SI (whole word), SUICID, HI (whole word), DEPRESS, ANXIET, PSYCH, ETOH, ALCOHOL, INTOX, OVERDOSE, OD (whole word), WITHDRAW, DRUG, AGITAT, HALLUCIN, HOMICID, MANIA, BIPOLAR, DETOX, HEROIN, COCAINE, OPIOID, BENZO |
| Chest pain | CHEST PAIN, CP (whole word), CHEST PRESSURE, CHEST TIGHT, CHEST DISCOMFORT |
| Dyspnea or respiratory | DYSPNEA, SOB (whole word), SHORTNESS OF BREATH, COUGH, RESP, WHEEZ, ASTHMA, COPD, HYPOXIA, BREATH, PNEUMONIA |
| Altered mental status or syncope | ALTERED, AMS (whole word), CONFUS, SYNCOPE, PRESYNCOPE, DIZZ, VERTIGO, LIGHTHEAD, UNRESPONSIVE, LETHARG, SOMNOLEN |
| Neurologic | HEADACHE, SEIZURE, STROKE, CVA (whole word), NUMB, TINGL, FACIAL DROOP, SLURRED, APHASIA, VISUAL, VISION, MIGRAINE, TIA (whole word), TREMOR |
| Abdominal or gastrointestinal | ABD, N/V, NAUSEA, VOMIT, DIARRHEA, EPIGASTRIC, FLANK, BRBPR, GI BLEED, HEMATEMESIS, MELENA, CONSTIPAT, RECTAL, BOWEL, GI (whole word), JAUNDICE, DYSPHAGIA |
| Fever or infection | FEVER, ILI (whole word), SEPSIS, CHILLS, CELLULITIS, ABSCESS, INFECT, COVID, FLU (whole word), SORE THROAT, PHARYNG, URI (whole word) |
| Cardiovascular | PALPITAT, HYPERTENS, HYPOTENS, TACHYCARD, BRADYCARD, AFIB, A-FIB, ARRHYTHM, EDEMA, SWELLING, DVT (whole word), CHF (whole word), HEART FAILURE, ICD (whole word), PACEMAKER, HTN (whole word) |
| Genitourinary or gynecologic | HEMATURIA, URIN, DYSURIA, UTI (whole word), VAGINAL, PREGNAN, PELVIC, TESTIC, PENILE, FOLEY, RENAL, KIDNEY, GU (whole word), MENSTRUAL, LABIA |
| Metabolic or laboratory | ABNORMAL LAB, ABNL LAB, HYPERGLYC, HYPOGLYC, HYPERKAL, HYPONATR, ANEMIA, DEHYDRAT, ELECTROLYTE, BLOOD SUGAR, HGB (whole word), INR (whole word), BS (whole word), GLUCOSE |
| Allergic or dermatologic | RASH, ALLERG, HIVES, ITCH, ANGIOEDEMA, URTICARIA, SKIN |
| Bleeding, other | EPISTAXIS, HEMOPTYSIS, BLEED, HEMORRH |
| Musculoskeletal pain | BACK PAIN, NECK PAIN, KNEE, LEG PAIN, FOOT, HIP, SHOULDER, HAND, ANKLE, WRIST, JOINT, EXTREMITY, PAIN, GOUT, SCIATICA |
| Weakness or functional decline | WEAK, FATIGUE, FAILURE TO THRIVE, GENERALIZED, MALAISE, UNABLE TO AMBULATE, DECONDITION, FTT (whole word) |

#### Top-coding of age

MIMIC-IV records age above 89 years at the anchor year as 91; all such stays fall in the 90 and older band (eTable 1, Panel C).

### Primary analyses

#### Discrimination

*Estimate.* Within each age band, the C statistic was the Mann-Whitney estimate, ties counted one half.

*Variance.* For each stay with the outcome, the placement value was the proportion of stays without the outcome ranked below it (ties one half). For each stay without the outcome, it was the proportion of stays with the outcome ranked above it. The influence contribution of a stay was its centered placement value divided by the number of stays in its outcome group. Contributions were summed within patient. The variance of the C statistic was the sum of the squared patient totals.

*Difference between two bands.* The variance of a band difference (the joint variance) was the sum of squared patient totals of each patient’s contribution to the older band minus that to the reference band. It allows for the 2794 patients with stays in both the 65 to 74 and the 18 to 64 bands. The other contrasts are unchanged, because the other bands share no patients with the 18 to 64 band.

*Use of the joint variance.* The analysis plan specified the sum of the two band variances, on the assumption that no patient had stays in both bands. Because that assumption failed for the 65 to 74 band, the joint variance was used instead, a change made after the primary results were known, for the primary outcome and for the band differences in the outcome components (eTable 4) and in the alternative outcome windows (eTable 8).

#### Transportability of level-specific risk

*Reference risk.* Transportability was assessed against the risk at each ESI level among stays aged 18 to 64 years. The levels were ESI 1, 2, and 3, and ESI 4-5 (levels 4 and 5 combined). For each level, the reference proportion was the observed proportion with the critical outcome at that level among stays aged 18 to 64 years. Each stay was assigned the logit of the reference proportion at its level as its reference linear predictor. The reference proportions were treated as fixed.

*Transport intercept and slope.* Within each band, the transport intercept was the intercept of a logistic model of the outcome with the reference linear predictor as an offset. The transport slope was the coefficient of a logistic model of the outcome on the reference linear predictor. Both were fitted by maximum likelihood with a cluster-robust (HC0) variance clustered on patient.

*Observed to expected ratio.* The ratio was the number of events divided by the sum of the reference proportions over the band’s stays.

#### Under-triage model

*Model.* Among stays with a critical outcome, logit P(ESI 3 to 5) = age band + s(heart rate) + s(systolic blood pressure) + s(respiratory rate) + s(oxygen saturation) + s(temperature) + chief-complaint category. Here s denotes a restricted cubic spline with knots at the 10th, 50th, and 90th percentiles of the analyzed stays.

*Age bands and variance.* Age band was coded with six levels (reference 18 to 64 years) and, in a second model, with three levels (18 to 64, 65 to 79, 80 years and older). Variances were cluster-robust (HC0) on patient.

*Sample size.* No sample size was calculated, the cohort being fixed; the adjusted under-triage model carried 31 parameters against the approximately 2900 under-triage events anticipated at design, more than 90 per parameter.

*Missing vital signs.* Missing vital signs were imputed by predictive mean matching in 20 data sets with 10 iterations. The imputation model included the under-triage indicator, age band, the five vital signs, chief-complaint category, arrival mode, and sex. Estimates were combined by Rubin’s rules.

### Additional analyses

1. Discrimination, transportability of level-specific risk, and under-triage by band in index stays only (eTable 7).
2. Age band by ESI level interaction in a logistic model of the critical outcome on the full analysis population, tested as one cluster-robust Wald statistic on the interaction terms (eTable 3, Panel C).
3. Discrimination and transportability of level-specific risk by band with the outcome window varied to ICU admission within 12 hours, within 48 hours, and with death within 7 days of ED arrival added from the recorded date of death (eTable 8).
4. The adjusted under-triage model with arrival mode, sex, and anchor-year group added.
5. The adjusted under-triage model in stays with complete vital signs.
6. The C statistic by anchor-year group in the reference and oldest-old bands, described without a test (eTable 9).

As a diagnostic of the clustering adjustment, standard errors that allow for repeat stays by the same patient were compared with standard errors that treat stays as independent. The ratio of the two is reported for the C statistic by age band and by anchor-year group, and for the age-band coefficients of the under-triage models (eTable 10).

The analyses below (outcome components, absolute performance, missing information and case mix) were specified after the primary results were known. The primary estimands, the cohort, and the outcome definition are unchanged.

#### Outcome components

In each age band and at 80 years and older, the following were counted: ICU admission within 24 hours of ED arrival; death in the ED or the linked hospitalization; death within 7 days of arrival from any source (the recorded date of death in MIMIC-IV); and the stays in the composite outcome with ICU admission without death. The C statistic of the ESI level was estimated for each of three outcomes taken alone: ICU admission within 24 hours, death in the ED or hospital, and death within 7 days. The method was the same as in the primary analysis, with the placement-value influence contributions summed within patient. The difference between each older band and the 18 to 64 band was estimated as well (eTable 4).

#### Absolute performance

ESI level 1 or 2 was taken as the positive test, and ESI level 1 to 3 as a second threshold. In each band and at 80 years and older, the following were estimated: sensitivity, specificity, positive and negative predictive values, the false-negative rate (1 minus sensitivity), and the number of critical-outcome stays below the threshold per 100 stays. Each proportion had a 95% confidence interval from an intercept-only logistic model with patient-cluster-robust variance (HC0), back-transformed from the logit scale. Where a proportion was based on zero events or on all events, this logit-scale interval cannot be computed. The counts are reported without an interval (eTable 5).

#### Bounds and scenarios for missing information

Both analyses bear on the primary contrast, the C statistic at 80 years and older minus the C statistic at 18 to 64 years (eTable 11).

*Stays without an ESI level. Bounds.* The 6986 excluded stays were added back, with the outcome derived as for included stays and the ESI level unknown. A band’s C statistic is highest when every added stay with the outcome is placed at ESI level 1 and every added stay without it at ESI level 5, and lowest under the reverse; these are exact extremes. The lower limit of the difference pairs the 80 years and older minimum with the 18 to 64 maximum. The upper limit pairs the 80 years and older maximum with the 18 to 64 minimum.

*Stays without an ESI level. Scenario.* In a plausible scenario, all added stays were placed at ESI level 1, because stays triaged without a recorded level were mostly ambulance arrivals with high outcome rates. This scenario is reported with a 95% confidence interval as in the primary analysis.

*Outcomes after transfer. Bounds.* Transfers are counted in the primary analysis as without the outcome. For the transfers in each band without a recorded outcome, any number at each ESI level (ESI 1, 2, and 3, and ESI 4-5) may be reassigned as having the outcome. Stays at the same level are tied for the C statistic, so it depends only on the number reassigned at each level. The search covered every combination of 0%, 10%, …, 100% of each level’s transfers. The minimum and maximum C statistic were recorded. The difference was bounded as for stays without an ESI level. These are extremes over that grid, not proven continuous extremes.

*Outcomes after transfer. Scenario.* In a second scenario, all transfers were counted as having the outcome. The result is reported with a 95% confidence interval.

*Intervals.* No interval is attached to a bound, because the bounds express missing information, not sampling error.

#### Case mix

*Stratified comparison.* Within each stratum of arrival mode (walk-in, ambulance, other or unknown) and, separately, of chief-complaint category, the C statistic was computed at 80 years and older and at 18 to 64 years, with the variance used in the primary analysis. The difference between them was taken. Differences were pooled by inverse variance across strata with at least 20 events in both groups. Strata excluded by that rule were counted. A patient can have stays in more than one stratum. The variance of the pooled difference therefore sums, within patient, the influence contributions to every stratum’s C statistics, multiplied by the fixed pooling weights.

*Reweighting.* A logistic model of membership in the 80 years and older group versus the 18 to 64 group was fitted on arrival mode, chief-complaint category, sex, and anchor-year group. Only pre-triage covariates were used: no ESI level, vital sign, or outcome. Stays aged 80 years and older were weighted by the inverse odds of membership, matching their covariate distribution to the 18 to 64 band. Its variance sums weighted placement-value influence contributions within patient, treating the weights as fixed. The difference against the unweighted 18 to 64 value is reported, with the effective sample size. Results are in eTable 6.

### Descriptive tabulations

Tabulated by age band: stays without an ESI level, with the critical outcome derived as for included stays (eTable 1, Panel B); ED disposition and deaths within 7 and 30 days of arrival, from the recorded date of death, among stays without a linked admission, with no outcome reassigned (eTable 13); and, for the five chief-complaint categories with a diagnostic analogue, the share of stays with a recorded first-listed ED diagnosis whose code falls in the matching ICD group (eTable 12).

## eTable 1. Excluded Stays Compared With the Analysis Population

**Panel A. Characteristics of excluded stays and the analysis population**

| Characteristic | Analysis population | Excluded, age not derivable^a^ | Excluded, ESI level missing |
| --- | --- | --- | --- |
| Stays, No. | 418 025 | 76 | 6986 |
| Patients, No. | 201 214 | 39 | 6808 |
| Age, median (IQR), y | 53 (35-69) | Not derivable | 55 (36-72) |
| Female sex, No. (%) | 226 976 (54.3) |  | 2893 (41.4) |
| Male sex, No. (%) | 191 049 (45.7) |  | 4093 (58.6) |
| Sex not recorded, No. (%) |  | 76 (100.0) |  |
| Walk-in arrival, No. (%) | 251 420 (60.1) | 44 (57.9) | 385 (5.5) |
| Ambulance arrival, No. (%) | 150 401 (36.0) | 26 (34.2) | 5325 (76.2) |
| Other or unknown arrival, No. (%) | 16 204 (3.9) | 6 (7.9) | 1276 (18.3) |
| ESI level 1, No. (%) | 24 014 (5.7) | 5 (6.6) |  |
| ESI level 2, No. (%) | 139 390 (33.3) | 21 (27.6) |  |
| ESI level 3, No. (%) | 225 024 (53.8) | 42 (55.3) |  |
| ESI level 4, No. (%) | 28 497 (6.8) | 7 (9.2) |  |
| ESI level 5, No. (%) | 1100 (0.3) | 0 (0.0) |  |
| ESI level not recorded, No. (%) |  | 1 (1.3) |  |
| Heart rate, median (IQR), beats/min | 84 (72-96); missing 10 313 | 84 (73-94); missing 4 | 87 (73-98); missing 6802 |
| Systolic blood pressure, median (IQR), mm Hg | 133 (120-148); missing 11 684 | 133 (117-141); missing 3 | 131 (118-146); missing 6807 |
| Respiratory rate, median (IQR), breaths/min | 18 (16-18); missing 13 588 | 16 (16-18); missing 5 | 18 (16-18); missing 6807 |
| Oxygen saturation, median (IQR), % | 99 (97-100); missing 13 926 | 99 (98-100); missing 5 | 99 (97-100); missing 6803 |
| Temperature, median (IQR), degrees F | 98.0 (97.5-98.6); missing 17 118 | 98.0 (97.6-98.5); missing 5 | 98.0 (97.6-98.5); missing 6816 |

**Panel B. Stays without and with a recorded ESI level, by age band**

| Age band | Stays, No.: without vs with ESI level | Without ESI level, % of band | Ambulance arrival, No. (%): without vs with | Admitted to hospital, No. (%): without vs with | Critical outcome, No. (%): without vs with^b^ |
| --- | --- | --- | --- | --- | --- |
| 18 to 64 y | 4482 vs 287 140 | 1.5 | 3345 (74.6) vs 83 277 (29.0) | 3151 (70.3) vs 110 093 (38.3) | 1896 (42.3) vs 11 209 (3.9) |
| 65 to 74 y | 1032 vs 58 711 | 1.7 | 780 (75.6) vs 25 283 (43.1) | 833 (80.7) vs 36 214 (61.7) | 630 (61.0) vs 5418 (9.2) |
| 75 to 79 y | 403 vs 22 443 | 1.8 | 324 (80.4) vs 11 124 (49.6) | 325 (80.6) vs 15 040 (67.0) | 251 (62.3) vs 2442 (10.9) |
| 80 to 84 y | 431 vs 19 687 | 2.1 | 344 (79.8) vs 10 983 (55.8) | 357 (82.8) vs 14 034 (71.3) | 266 (61.7) vs 2399 (12.2) |
| 85 to 89 y | 354 vs 16 121 | 2.1 | 299 (84.5) vs 10 047 (62.3) | 289 (81.6) vs 11 834 (73.4) | 223 (63.0) vs 2051 (12.7) |
| 90 y and older | 284 vs 13 923 | 2.0 | 233 (82.0) vs 9687 (69.6) | 236 (83.1) vs 10 584 (76.0) | 188 (66.2) vs 1888 (13.6) |
| 80 y and older | 1069 vs 49 731 | 2.1 | 876 (81.9) vs 30 717 (61.8) | 882 (82.5) vs 36 452 (73.3) | 677 (63.3) vs 6338 (12.7) |

**Panel C. Top-coding of age, by age band^c^**

| Age band | Stays, No. | Stays of top-coded patients, No. | Top-coded patients, No. | Stays with derived age of 91 y, No.^d^ |
| --- | --- | --- | --- | --- |
| 90 y and older | 13 923 | 7817 | 4211 | 12 165 |
| 80 y and older | 49 731 | 7817 | 4211 | 12 165 |

Abbreviations: ESI, Emergency Severity Index; IQR, interquartile range.

No stays were excluded for age under 18 years. Percentages in Panel A are of the column’s stays; admission, outcomes, and chief-complaint categories for the analysis population are in Table 1 and eTable 2.

^a^Age could not be derived, so no age-dependent eligibility check was applied; only the variables shown are available for this group.

^b^The critical outcome was derived for stays without a recorded ESI level exactly as for stays with one.

^c^Top-coded patients are those whose age at the anchor year was recorded as 91, because MIMIC-IV records age above 89 years at the anchor year as 91. Each of the bands 18 to 64, 65 to 74, 75 to 79, 80 to 84, and 85 to 89 y had 0 in all three top-coding columns.

^d^Derived age at arrival is capped at 91 years, so this count includes stays of patients who were not top-coded.

## eTable 2. Chief-Complaint Category, Triage Vital Signs, and Missingness by Age Band

**Panel A. Chief-complaint category, No. (%)**

| Chief-complaint category | Overall | 18 to 64 y | 65 to 74 y | 75 to 79 y | 80 to 84 y | 85 to 89 y | 90 y and older | 80 y and older |
| --- | --- | --- | --- | --- | --- | --- | --- | --- |
| Stays, No. | 418 025 | 287 140 | 58 711 | 22 443 | 19 687 | 16 121 | 13 923 | 49 731 |
| Trauma or injury | 55 799 (13.3) | 36 686 (12.8) | 6940 (11.8) | 2950 (13.1) | 3053 (15.5) | 2984 (18.5) | 3186 (22.9) | 9223 (18.5) |
| Psychiatric or substance | 28 533 (6.8) | 26 462 (9.2) | 1368 (2.3) | 283 (1.3) | 148 (0.8) | 134 (0.8) | 138 (1.0) | 420 (0.8) |
| Chest pain | 30 630 (7.3) | 20 349 (7.1) | 5212 (8.9) | 1768 (7.9) | 1391 (7.1) | 1086 (6.7) | 824 (5.9) | 3301 (6.6) |
| Dyspnea or respiratory | 29 611 (7.1) | 14 647 (5.1) | 6111 (10.4) | 2646 (11.8) | 2343 (11.9) | 1994 (12.4) | 1870 (13.4) | 6207 (12.5) |
| Altered mental status or syncope | 25 651 (6.1) | 13 595 (4.7) | 5024 (8.6) | 2133 (9.5) | 1951 (9.9) | 1637 (10.2) | 1311 (9.4) | 4899 (9.9) |
| Neurologic | 22 428 (5.4) | 17 011 (5.9) | 2619 (4.5) | 973 (4.3) | 796 (4.0) | 609 (3.8) | 420 (3.0) | 1825 (3.7) |
| Abdominal or gastrointestinal | 74 146 (17.7) | 54 384 (18.9) | 9752 (16.6) | 3365 (15.0) | 2875 (14.6) | 2107 (13.1) | 1663 (11.9) | 6645 (13.4) |
| Fever or infection | 17 278 (4.1) | 12 922 (4.5) | 2271 (3.9) | 778 (3.5) | 592 (3.0) | 407 (2.5) | 308 (2.2) | 1307 (2.6) |
| Cardiovascular | 15 863 (3.8) | 9292 (3.2) | 2957 (5.0) | 1133 (5.0) | 1039 (5.3) | 786 (4.9) | 656 (4.7) | 2481 (5.0) |
| Genitourinary or gynecologic | 11 354 (2.7) | 7827 (2.7) | 1512 (2.6) | 675 (3.0) | 542 (2.8) | 432 (2.7) | 366 (2.6) | 1340 (2.7) |
| Metabolic or laboratory | 7809 (1.9) | 4067 (1.4) | 1692 (2.9) | 679 (3.0) | 597 (3.0) | 453 (2.8) | 321 (2.3) | 1371 (2.8) |
| Allergic or dermatologic | 5469 (1.3) | 4655 (1.6) | 464 (0.8) | 127 (0.6) | 90 (0.5) | 85 (0.5) | 48 (0.3) | 223 (0.4) |
| Bleeding, other | 2538 (0.6) | 1312 (0.5) | 478 (0.8) | 253 (1.1) | 197 (1.0) | 175 (1.1) | 123 (0.9) | 495 (1.0) |
| Musculoskeletal pain | 46 646 (11.2) | 35 378 (12.3) | 5693 (9.7) | 1900 (8.5) | 1521 (7.7) | 1201 (7.4) | 953 (6.8) | 3675 (7.4) |
| Weakness or functional decline | 7072 (1.7) | 3115 (1.1) | 1407 (2.4) | 699 (3.1) | 717 (3.6) | 588 (3.6) | 546 (3.9) | 1851 (3.7) |
| Other or not recorded | 37 198 (8.9) | 25 438 (8.9) | 5211 (8.9) | 2081 (9.3) | 1835 (9.3) | 1443 (9.0) | 1190 (8.5) | 4468 (9.0) |

**Panel B. Triage vital signs, median (IQR), with the number missing^a^**

| Vital sign | Overall | 18 to 64 y | 65 to 74 y | 75 to 79 y | 80 to 84 y | 85 to 89 y | 90 y and older | 80 y and older |
| --- | --- | --- | --- | --- | --- | --- | --- | --- |
| Heart rate, beats/min | 84 (72-96); missing 10 313 | 86 (74-98); missing 5808 | 80 (70-93); missing 1731 | 80 (68-91); missing 734 | 79 (68-90); missing 742 | 78 (67-89); missing 656 | 77 (67-89); missing 642 | 78 (68-89); missing 2040 |
| Systolic blood pressure, mm Hg | 133 (120-148); missing 11 684 | 131 (119-145); missing 6878 | 138 (122-154); missing 1890 | 139 (123-156); missing 818 | 139 (122-157); missing 774 | 140 (122-158); missing 677 | 140 (123-159); missing 647 | 140 (122-158); missing 2098 |
| Respiratory rate, breaths/min | 18 (16-18); missing 13 588 | 18 (16-18); missing 7889 | 18 (16-18); missing 2203 | 18 (16-18); missing 939 | 18 (16-18); missing 959 | 18 (16-18); missing 809 | 18 (16-18); missing 789 | 18 (16-18); missing 2557 |
| Oxygen saturation, % | 99 (97-100); missing 13 926 | 99 (98-100); missing 8060 | 98 (97-100); missing 2285 | 98 (97-100); missing 975 | 98 (96-100); missing 946 | 98 (96-99); missing 852 | 98 (96-99); missing 808 | 98 (96-99); missing 2606 |
| Temperature, degrees F | 98.0 (97.5-98.6); missing 17 118 | 98.0 (97.6-98.6); missing 10 028 | 98.0 (97.5-98.5); missing 2712 | 98.0 (97.5-98.5); missing 1197 | 98.0 (97.5-98.5); missing 1192 | 98.0 (97.5-98.5); missing 1008 | 98.0 (97.5-98.4); missing 981 | 98.0 (97.5-98.5); missing 3181 |

**Panel C. Missing triage vital signs by age band and critical outcome, %: stays with vs without the critical outcome^b^**

| Age band | Events, No. | Heart rate | Systolic blood pressure | Respiratory rate | Oxygen saturation | Temperature | Any of the five |
| --- | --- | --- | --- | --- | --- | --- | --- |
| 18 to 64 y | 11 209 | 18.1 vs 1.4 | 18.3 vs 1.7 | 20.1 vs 2.0 | 19.9 vs 2.1 | 23.2 vs 2.7 | 26.2 vs 4.7 |
| 65 to 74 y | 5418 | 17.7 vs 1.4 | 17.8 vs 1.7 | 19.1 vs 2.2 | 19.7 vs 2.3 | 22.5 vs 2.8 | 25.8 vs 4.8 |
| 75 to 79 y | 2442 | 18.3 vs 1.4 | 18.2 vs 1.9 | 19.8 vs 2.3 | 20.5 vs 2.4 | 24.2 vs 3.0 | 27.0 vs 5.2 |
| 80 to 84 y | 2399 | 18.5 vs 1.7 | 18.6 vs 1.9 | 20.9 vs 2.6 | 20.4 vs 2.6 | 24.5 vs 3.5 | 27.7 vs 5.8 |
| 85 to 89 y | 2051 | 19.7 vs 1.8 | 19.5 vs 2.0 | 20.6 vs 2.7 | 21.6 vs 2.9 | 25.0 vs 3.5 | 27.8 vs 6.0 |
| 90 y and older | 1888 | 20.1 vs 2.2 | 19.5 vs 2.3 | 21.9 vs 3.1 | 21.9 vs 3.3 | 26.0 vs 4.1 | 30.0 vs 6.5 |
| 80 y and older | 6338 | 19.4 vs 1.9 | 19.1 vs 2.0 | 21.1 vs 2.8 | 21.2 vs 2.9 | 25.1 vs 3.7 | 28.4 vs 6.1 |
| All ages | 25 407 | 18.3 vs 1.4 | 18.4 vs 1.8 | 20.1 vs 2.2 | 20.3 vs 2.2 | 23.6 vs 2.8 | 26.8 vs 4.9 |

**Panel D. Plausible ranges and values set to missing**

| Vital sign | Plausible range | Missing as recorded, No. | Implausible values set to missing, No. | Missing after, No. |
| --- | --- | --- | --- | --- |
| Heart rate, beats/min | 20-300 | 10 284 | 29 | 10 313 |
| Systolic blood pressure, mm Hg | 40-300 | 11 481 | 203 | 11 684 |
| Respiratory rate, breaths/min | 4-80 | 13 541 | 47 | 13 588 |
| Oxygen saturation, % | 50-100 | 13 788 | 138 | 13 926 |
| Temperature, degrees F | 80-110 | 16 594 | 524 | 17 118 |

Abbreviations: IQR, interquartile range.

^a^Values outside the plausible ranges (Panel D) were set to missing before imputation; missing counts include values missing as recorded and values set to missing as implausible.

^b^Percentage of stays with a missing value, among stays with and among stays without the critical outcome in the band. Any of the five is the percentage of stays missing one or more of the five vital signs. For all ages, stays missing each vital sign numbered, with and without the critical outcome (of 25 407 and 392 618 stays): heart rate, 4660 and 5653; systolic blood pressure, 4679 and 7005; respiratory rate, 5108 and 8480; oxygen saturation, 5148 and 8778; temperature, 5998 and 11 120; any of the five, 6801 and 19 111.

## eTable 3. Reference Risk, Observed to Expected Ratio by ESI Level, and Age Band by ESI Level Interaction Test

**Panel A. Reference risk by ESI level (18 to 64 y)**

| ESI level | Reference stays, No. | Reference events, No. | Reference risk, %^a^ |
| --- | --- | --- | --- |
| ESI 1 | 13 070 | 3714 | 28.4 |
| ESI 2 | 82 091 | 5930 | 7.2 |
| ESI 3 | 165 700 | 1553 | 0.9 |
| ESI 4-5 | 26 279 | 12 | <0.1 |

**Panel B. Observed to expected ratio by ESI level within each older age band^b^**

| Age band | ESI 1: events/stays (observed risk, %) | ESI 1: O/E | ESI 2: events/stays (observed risk, %) | ESI 2: O/E | ESI 3: events/stays (observed risk, %) | ESI 3: O/E | ESI 4-5: events/stays (observed risk, %)^c^ | ESI 4-5: O/E^c^ |
| --- | --- | --- | --- | --- | --- | --- | --- | --- |
| 65 to 74 y | 1798/4337 (41.5) | 1.46 | 2999/24 320 (12.3) | 1.71 | 619/27 968 (2.2) | 2.36 | 2/2086 (0.1) | 2.10 |
| 75 to 79 y | 832/1800 (46.2) | 1.63 | 1354/9927 (13.6) | 1.89 | 255/10 162 (2.5) | 2.68 | 1/554 (0.2) | 3.95 |
| 80 to 84 y | 844/1817 (46.5) | 1.63 | 1302/9015 (14.4) | 2.00 | 253/8518 (3.0) | 3.17 | 0/337 (0.0) | 0.00 |
| 85 to 89 y | 706/1520 (46.4) | 1.63 | 1106/7499 (14.7) | 2.04 | 239/6890 (3.5) | 3.70 | 0/212 (0.0) | 0.00 |
| 90 y and older | 695/1470 (47.3) | 1.66 | 980/6538 (15.0) | 2.08 | 212/5786 (3.7) | 3.91 | 1/129 (0.8) | 16.98 |
| 80 y and older | 2245/4807 (46.7) | 1.64 | 3388/23 052 (14.7) | 2.03 | 704/21 194 (3.3) | 3.54 | 1/678 (0.1) | 3.23 |

**Panel C. Age band by ESI level interaction test**

| Test | Wald statistic | Degrees of freedom | *P* value |
| --- | --- | --- | --- |
| Age band by ESI level^d^ | 1075.6 | 20 | *P* < .001 |

Abbreviations: ESI, Emergency Severity Index; O/E, observed to expected ratio.

Expected risk at every level in every band is the reference risk in Panel A; the ratio is 1.00 in the reference band (18 to 64 y) at every level.

^a^Observed risk of the critical outcome at each ESI level among stays 18 to 64 years old.

^b^Events/stays at each ESI level; O/E is the observed risk divided by the Panel A reference risk.

^c^The reference band had 12 events among 26 279 stays at ESI 4-5 and the older bands 0 to 2 events per band, so ratios at ESI 4-5 are not interpreted.

^d^Model with an age band by ESI level interaction term compared with the main-effects model, over the full analysis population; the 20 degrees of freedom are 5 age-band contrasts times 4 ESI-level contrasts.

## eTable 4. Outcome Components and Discrimination of the ESI Level for Each Component by Age Band

**Panel A. Counts of outcome components by age band, No.**

| Age band | Stays | Composite critical outcome^a^ | ICU admission within 24 hours | Death in ED or hospital | Death within 7 days of ED arrival, any source^b^ | Composite stays with ICU admission without death, % |
| --- | --- | --- | --- | --- | --- | --- |
| 18 to 64 y | 287 140 | 11 209 | 10 796 | 1094 | 680 | 90.2 |
| 65 to 74 y | 58 711 | 5418 | 5056 | 874 | 546 | 83.9 |
| 75 to 79 y | 22 443 | 2442 | 2280 | 466 | 310 | 80.9 |
| 80 to 84 y | 19 687 | 2399 | 2200 | 520 | 358 | 78.3 |
| 85 to 89 y | 16 121 | 2051 | 1834 | 504 | 393 | 75.4 |
| 90 y and older | 13 923 | 1888 | 1622 | 575 | 517 | 69.5 |
| 80 y and older | 49 731 | 6338 | 5656 | 1599 | 1268 | 74.8 |

**Panel B. C statistic of the ESI level for each component taken alone, C statistic (95% CI) and difference vs 18 to 64 y (95% CI)**

| Age band | ICU admission within 24 hours: C statistic | Difference | Death in ED or hospital: C statistic | Difference | Death within 7 days: C statistic | Difference |
| --- | --- | --- | --- | --- | --- | --- |
| 18 to 64 y | 0.822 (0.818-0.825) |  | 0.813 (0.802-0.825) |  | 0.834 (0.819-0.849) |  |
| 65 to 74 y | 0.782 (0.776-0.788) | -0.040 (-0.047 to -0.033) | 0.740 (0.725-0.756) | -0.073 (-0.092 to -0.054) | 0.775 (0.757-0.793) | -0.059 (-0.082 to -0.035) |
| 75 to 79 y | 0.774 (0.765-0.783) | -0.047 (-0.057 to -0.038) | 0.770 (0.751-0.789) | -0.043 (-0.066 to -0.021) | 0.791 (0.769-0.814) | -0.043 (-0.069 to -0.016) |
| 80 to 84 y | 0.766 (0.757-0.775) | -0.056 (-0.066 to -0.046) | 0.747 (0.727-0.767) | -0.067 (-0.090 to -0.044) | 0.769 (0.745-0.793) | -0.065 (-0.093 to -0.037) |
| 85 to 89 y | 0.756 (0.746-0.767) | -0.065 (-0.076 to -0.054) | 0.731 (0.711-0.752) | -0.082 (-0.105 to -0.059) | 0.743 (0.720-0.767) | -0.091 (-0.119 to -0.063) |
| 90 y and older | 0.757 (0.746-0.768) | -0.064 (-0.076 to -0.052) | 0.715 (0.695-0.735) | -0.098 (-0.122 to -0.075) | 0.731 (0.710-0.752) | -0.103 (-0.129 to -0.078) |
| 80 y and older | 0.760 (0.754-0.766) | -0.061 (-0.068 to -0.054) | 0.731 (0.720-0.743) | -0.082 (-0.098 to -0.065) | 0.747 (0.734-0.760) | -0.087 (-0.107 to -0.068) |

Abbreviations: CI, confidence interval; ED, emergency department; ESI, Emergency Severity Index; ICU, intensive care unit. Event counts in Panel B are the component counts in Panel A.

^a^Death in the ED or the linked hospitalization, or ICU admission within 24 hours of ED arrival.

^b^From the recorded date of death, from any source; not part of the composite critical outcome.

## eTable 5. Absolute Performance of the ESI Level at Two Thresholds by Age Band

**Panel A. ESI 1 or 2 as the positive test**

| Age band | Sensitivity, % (95% CI) | Specificity, % (95% CI) | PPV, % (95% CI) | NPV, % (95% CI) | False-negative rate, % (95% CI) | Critical-outcome stays at ESI 3-5 per 100 stays (95% CI) |
| --- | --- | --- | --- | --- | --- | --- |
| 18 to 64 y | 86.0 (85.3-86.7) | 69.0 (68.8-69.3) | 10.1 (9.9-10.4) | 99.2 (99.1-99.2) | 14.0 (13.3-14.7) | 0.55 (0.52-0.58) |
| 65 to 74 y | 88.5 (87.6-89.4) | 55.2 (54.6-55.8) | 16.7 (16.2-17.3) | 97.9 (97.8-98.1) | 11.5 (10.6-12.4) | 1.06 (0.97-1.15) |
| 75 to 79 y | 89.5 (88.2-90.7) | 52.3 (51.4-53.2) | 18.6 (17.9-19.5) | 97.6 (97.3-97.9) | 10.5 (9.3-11.8) | 1.14 (1.01-1.29) |
| 80 to 84 y | 89.5 (88.1-90.6) | 49.8 (48.8-50.7) | 19.8 (19.0-20.6) | 97.1 (96.8-97.5) | 10.5 (9.4-11.9) | 1.29 (1.14-1.45) |
| 85 to 89 y | 88.3 (86.9-89.7) | 48.8 (47.8-49.7) | 20.1 (19.2-21.0) | 96.6 (96.2-97.0) | 11.7 (10.3-13.1) | 1.48 (1.31-1.68) |
| 90 y and older | 88.7 (87.2-90.1) | 47.4 (46.2-48.5) | 20.9 (20.0-21.9) | 96.4 (95.9-96.9) | 11.3 (9.9-12.8) | 1.53 (1.34-1.75) |
| 80 y and older | 88.9 (88.1-89.6) | 48.8 (48.2-49.4) | 20.2 (19.7-20.7) | 96.8 (96.5-97.0) | 11.1 (10.4-11.9) | 1.42 (1.32-1.53) |

**Panel B. ESI 1, 2, or 3 as the positive test**

| Age band | Sensitivity, % (95% CI) | Specificity, % (95% CI) | PPV, % (95% CI) | NPV, % (95% CI) | False-negative rate, % (95% CI) |
| --- | --- | --- | --- | --- | --- |
| 18 to 64 y | 99.9 (99.8-99.9) | 9.5 (9.4-9.7) | 4.3 (4.2-4.4) | 100.0 (99.9-100.0) | 0.1 (0.1-0.2) |
| 65 to 74 y | 100.0 (99.9-100.0) | 3.9 (3.7-4.1) | 9.6 (9.3-9.9) | 99.9 (99.6-100.0) | <0.1 (0.0-0.1) |
| 75 to 79 y | 100.0 (99.7-100.0) | 2.8 (2.4-3.1) | 11.2 (10.7-11.7) | 99.8 (98.7-100.0) | <0.1 (0.0-0.3) |
| 80 to 84 y | 2399/2399^a^ | 1.9 (1.7-2.2) | 12.4 (11.9-12.9) | 337/337^a^ | 0/2399^a^ |
| 85 to 89 y | 2051/2051^a^ | 1.5 (1.3-1.7) | 12.9 (12.3-13.5) | 212/212^a^ | 0/2051^a^ |
| 90 y and older | 99.9 (99.6-100.0) | 1.1 (0.9-1.3) | 13.7 (13.0-14.3) | 99.2 (94.7-99.9) | 0.1 (0.0-0.4) |
| 80 y and older | 100.0 (99.9-100.0) | 1.6 (1.4-1.7) | 12.9 (12.6-13.3) | 99.9 (99.0-100.0) | <0.1 (0.0-0.1) |

Abbreviations: CI, confidence interval; ESI, Emergency Severity Index; NPV, negative predictive value; PPV, positive predictive value. The false-negative rate is 100 minus sensitivity. Intervals are patient-cluster-robust.

^a^Interval not estimable because all or none of the stays in the cell had the event; events/stays in the cell are shown.

## eTable 6. Case Mix: Stratified and Reweighted Comparisons of the C Statistic, 80 y and Older Versus 18 to 64 y

**Panel A. Within-stratum difference in the C statistic (80 y and older minus 18 to 64 y)^a^**

| Stratum | Events, 80 y and older | Events, 18 to 64 y | Difference (95% CI) |
| --- | --- | --- | --- |
| **Arrival mode** |  |  |  |
| Walk-in | 927 | 2891 | -0.084 (-0.102 to -0.067) |
| Ambulance | 4561 | 6312 | -0.016 (-0.025 to -0.007) |
| Other or unknown | 850 | 2006 | -0.099 (-0.119 to -0.079) |
| **Chief-complaint category** |  |  |  |
| Trauma or injury | 747 | 802 | -0.144 (-0.164 to -0.124) |
| Psychiatric or substance^b^ | 13 | 631 | NE |
| Chest pain | 262 | 540 | -0.062 (-0.095 to -0.028) |
| Dyspnea or respiratory | 1354 | 1593 | -0.067 (-0.084 to -0.050) |
| Altered mental status or syncope | 681 | 934 | -0.077 (-0.100 to -0.054) |
| Neurologic | 390 | 948 | -0.030 (-0.057 to -0.003) |
| Abdominal or gastrointestinal | 784 | 1819 | -0.064 (-0.084 to -0.043) |
| Fever or infection | 199 | 544 | -0.043 (-0.077 to -0.008) |
| Cardiovascular | 335 | 524 | -0.035 (-0.061 to -0.008) |
| Genitourinary or gynecologic | 59 | 67 | -0.092 (-0.179 to -0.005) |
| Metabolic or laboratory | 153 | 432 | -0.087 (-0.135 to -0.039) |
| Allergic or dermatologic^b^ | 4 | 80 | NE |
| Bleeding, other | 67 | 124 | -0.037 (-0.095 to 0.021) |
| Musculoskeletal pain | 102 | 183 | -0.081 (-0.135 to -0.026) |
| Weakness or functional decline | 236 | 252 | -0.069 (-0.114 to -0.023) |
| Other or not recorded | 952 | 1736 | -0.055 (-0.071 to -0.039) |

**Panel B. Differences pooled across strata by inverse variance^c^, and reweighting of stays aged 80 y and older to the 18 to 64 y case mix**

| Analysis | Strata included / excluded, No. | C statistic, 80 y and older | C statistic, 18 to 64 y | Difference (95% CI) | Effective sample size, 80 y and older |
| --- | --- | --- | --- | --- | --- |
| Pooled, arrival mode | 3 / 0 |  |  | -0.040 (-0.048 to -0.033) |  |
| Pooled, chief-complaint category | 14 / 2 |  |  | -0.069 (-0.076 to -0.062) |  |
| Primary analysis (unweighted) |  | 0.754 | 0.818 | -0.065 (-0.072 to -0.058) | 49 731 |
| Reweighted to the 18 to 64 y case mix^d^ |  | 0.760 | 0.818 | -0.058 (-0.071 to -0.046) | 10 389 |

Weights, stays aged 80 y and older: minimum 0.58, median 3.14, 99th percentile 28.89, maximum 296.76.

**Panel C. Covariate balance, % of stays**

| Covariate and level | 18 to 64 y | 80 y and older, unweighted | 80 y and older, weighted |
| --- | --- | --- | --- |
| Arrival mode: walk-in | 67.0 | 34.7 | 68.6 |
| Arrival mode: ambulance | 29.0 | 61.8 | 27.3 |
| Arrival mode: other or unknown | 4.0 | 3.6 | 4.1 |
| Complaint: trauma or injury | 12.8 | 18.5 | 10.8 |
| Complaint: psychiatric or substance | 9.2 | 0.8 | 12.7 |
| Complaint: dyspnea or respiratory | 5.1 | 12.5 | 5.2 |
| Complaint: altered mental status or syncope | 4.7 | 9.9 | 5.0 |
| Complaint: abdominal or gastrointestinal | 18.9 | 13.4 | 18.4 |
| Complaint: musculoskeletal pain | 12.3 | 7.4 | 11.0 |
| Sex: female | 53.9 | 60.4 | 52.7 |
| Anchor-year group 2008-2010 | 25.7 | 37.9 | 27.3 |
| Anchor-year group 2011-2013 | 28.1 | 26.6 | 28.5 |
| Anchor-year group 2014-2016 | 26.8 | 20.9 | 26.3 |
| Anchor-year group 2017-2019 | 19.4 | 14.6 | 18.0 |

Abbreviations: CI, confidence interval; ESI, Emergency Severity Index; NE, not estimated. Panel C shows the chief-complaint categories with the largest differences between age groups; the other categories were also in the weighting model.

^a^Difference in the C statistic at 80 y and older minus 18 to 64 y within each stratum.

^b^Fewer than 20 events in one or both age groups; excluded from pooling.

^c^Pooled over strata with at least 20 events in both age groups.

^d^Weights were inverse odds from a logistic model of membership in the 80 y and older group versus the 18 to 64 y group on arrival mode, chief-complaint category, sex, and anchor-year group; no ESI level, vital sign, or outcome entered the model.

## eFigure 1. Age Difference in the C Statistic Across the Primary and Additional Analyses

Points are the difference in the C statistic of the Emergency Severity Index (ESI) level, 80 y and older minus 18 to 64 y, with 95% confidence intervals that account for repeat stays by the same patient. The dashed line marks the primary estimate (-0.065); the solid line marks no difference. Exact values are in eTables 4, 6, 7, 8, and 11. The two analyses of missing information give bounds, not intervals, and are listed without a mark: -0.167 to 0.082 over every ESI assignment of stays without an ESI level, and -0.089 to -0.037 with transfers reassigned to the outcome in 10% steps within each ESI level (eTable 11).

## eTable 7. Index Stays Only (First ED Stay of Each Patient)

**Panel A. Discrimination and transportability of level-specific risk by age band**

| Age band | Stays | Events | C statistic (95% CI) | Difference vs 18 to 64 y (95% CI) | Transport intercept (95% CI) | Transport slope (95% CI) | Observed to expected ratio |
| --- | --- | --- | --- | --- | --- | --- | --- |
| 18 to 64 y | 147 894 | 6041 | 0.837 (0.832-0.841) |  | 0.00 (-0.03 to 0.03) | 1.00 (0.98-1.02) | 1.00 |
| 65 to 74 y | 24 780 | 2688 | 0.778 (0.770-0.786) | -0.059 (-0.067 to -0.050) | 0.71 (0.67-0.76) | 0.90 (0.86-0.94) | 1.77 |
| 75 to 79 y | 9272 | 1261 | 0.770 (0.758-0.781) | -0.067 (-0.080 to -0.055) | 0.90 (0.84-0.97) | 0.92 (0.86-0.99) | 2.03 |
| 80 to 84 y | 8016 | 1249 | 0.762 (0.749-0.774) | -0.075 (-0.088 to -0.062) | 0.98 (0.91-1.04) | 0.90 (0.83-0.96) | 2.11 |
| 85 to 89 y | 6211 | 998 | 0.754 (0.739-0.768) | -0.083 (-0.098 to -0.068) | 1.03 (0.95-1.10) | 0.87 (0.80-0.95) | 2.19 |
| 90 y and older | 5041 | 921 | 0.733 (0.717-0.749) | -0.103 (-0.120 to -0.087) | 1.09 (1.01-1.17) | 0.77 (0.69-0.84) | 2.26 |
| 80 y and older | 19 268 | 3168 | 0.751 (0.743-0.760) | -0.085 (-0.094 to -0.076) | 1.02 (0.98-1.07) | 0.85 (0.81-0.89) | 2.18 |

**Panel B. Under-triage odds ratio by age band**

| Age band | Unadjusted OR (95% CI) | Adjusted OR, multiple imputation (95% CI)^a^ | Complete case OR (95% CI)^b^ |
| --- | --- | --- | --- |
| **Six age bands relative to 18 to 64 y** |  |  |  |
| 65 to 74 y | 0.84 (0.72-0.97) | 0.81 (0.69-0.96) | 0.77 (0.65-0.91) |
| 75 to 79 y | 0.74 (0.59-0.92) | 0.75 (0.59-0.94) | 0.74 (0.59-0.93) |
| 80 to 84 y | 0.71 (0.57-0.88) | 0.72 (0.57-0.91) | 0.69 (0.54-0.89) |
| 85 to 89 y | 0.84 (0.67-1.06) | 0.91 (0.72-1.16) | 0.86 (0.67-1.11) |
| 90 y and older | 0.92 (0.73-1.16) | 1.02 (0.80-1.30) | 1.03 (0.80-1.33) |
| **Two age bands relative to 18 to 64 y** |  |  |  |
| 65 to 79 y | 0.80 (0.70-0.92) | 0.79 (0.69-0.92) | 0.76 (0.65-0.88) |
| 80 y and older | 0.81 (0.70-0.94) | 0.86 (0.74-1.01) | 0.84 (0.71-0.99) |

Abbreviations: CI, confidence interval; ED, emergency department; OR, odds ratio.

^a^Adjusted for triage vital signs and chief-complaint category, combined across 20 imputed data sets.

^b^Restricted to stays with complete triage vital signs and chief-complaint category.

## eTable 8. Alternative Outcome Windows: Discrimination and Transportability by Age Band

| Age band | Stays | Events | C statistic (95% CI) | Difference vs 18 to 64 y (95% CI)^a^ | Transport intercept (95% CI) | Transport slope (95% CI) | Observed to expected ratio |
| --- | --- | --- | --- | --- | --- | --- | --- |
| **ICU admission within 12 hours** |  |  |  |  |  |  |  |
| 18 to 64 y | 287 140 | 10 195 | 0.829 (0.825-0.833) |  | 0.00 (-0.03 to 0.03) | 1.00 (0.98-1.02) | 1.00 |
| 65 to 74 y | 58 711 | 5006 | 0.784 (0.778-0.790) | -0.045 (-0.052 to -0.038) | 0.63 (0.59-0.67) | 0.93 (0.90-0.96) | 1.67 |
| 75 to 79 y | 22 443 | 2261 | 0.780 (0.771-0.788) | -0.049 (-0.059 to -0.040) | 0.76 (0.71-0.82) | 0.95 (0.91-1.00) | 1.86 |
| 80 to 84 y | 19 687 | 2197 | 0.766 (0.756-0.775) | -0.063 (-0.073 to -0.053) | 0.81 (0.76-0.86) | 0.89 (0.85-0.94) | 1.91 |
| 85 to 89 y | 16 121 | 1896 | 0.757 (0.747-0.767) | -0.072 (-0.083 to -0.061) | 0.86 (0.80-0.91) | 0.86 (0.81-0.91) | 1.98 |
| 90 y and older | 13 923 | 1767 | 0.755 (0.744-0.766) | -0.074 (-0.086 to -0.062) | 0.90 (0.84-0.95) | 0.85 (0.80-0.90) | 2.02 |
| 80 y and older | 49 731 | 5860 | 0.760 (0.754-0.766) | -0.069 (-0.076 to -0.062) | 0.85 (0.82-0.88) | 0.87 (0.84-0.90) | 1.96 |
| **ICU admission within 48 hours** |  |  |  |  |  |  |  |
| 18 to 64 y | 287 140 | 11 806 | 0.812 (0.808-0.816) |  | 0.00 (-0.02 to 0.02) | 1.00 (0.98-1.02) | 1.00 |
| 65 to 74 y | 58 711 | 5716 | 0.768 (0.762-0.774) | -0.044 (-0.051 to -0.037) | 0.63 (0.60-0.67) | 0.93 (0.90-0.95) | 1.68 |
| 75 to 79 y | 22 443 | 2566 | 0.762 (0.753-0.771) | -0.050 (-0.060 to -0.041) | 0.77 (0.72-0.82) | 0.93 (0.89-0.98) | 1.85 |
| 80 to 84 y | 19 687 | 2506 | 0.756 (0.747-0.765) | -0.056 (-0.066 to -0.046) | 0.82 (0.77-0.87) | 0.91 (0.87-0.96) | 1.92 |
| 85 to 89 y | 16 121 | 2151 | 0.741 (0.731-0.752) | -0.071 (-0.082 to -0.060) | 0.87 (0.81-0.92) | 0.85 (0.81-0.90) | 1.98 |
| 90 y and older | 13 923 | 1953 | 0.745 (0.734-0.756) | -0.067 (-0.079 to -0.056) | 0.88 (0.82-0.93) | 0.86 (0.81-0.92) | 1.98 |
| 80 y and older | 49 731 | 6610 | 0.748 (0.742-0.754) | -0.064 (-0.071 to -0.057) | 0.85 (0.82-0.88) | 0.88 (0.85-0.91) | 1.96 |
| **ICU admission within 24 hours or death within 7 days** |  |  |  |  |  |  |  |
| 18 to 64 y | 287 140 | 11 303 | 0.817 (0.813-0.821) |  | 0.00 (-0.03 to 0.03) | 1.00 (0.98-1.02) | 1.00 |
| 65 to 74 y | 58 711 | 5494 | 0.773 (0.767-0.779) | -0.044 (-0.051 to -0.037) | 0.63 (0.60-0.67) | 0.93 (0.90-0.96) | 1.68 |
| 75 to 79 y | 22 443 | 2482 | 0.769 (0.760-0.778) | -0.048 (-0.058 to -0.039) | 0.77 (0.72-0.82) | 0.95 (0.90-0.99) | 1.86 |
| 80 to 84 y | 19 687 | 2434 | 0.759 (0.750-0.768) | -0.058 (-0.068 to -0.048) | 0.83 (0.78-0.88) | 0.91 (0.86-0.95) | 1.94 |
| 85 to 89 y | 16 121 | 2108 | 0.748 (0.737-0.758) | -0.070 (-0.080 to -0.059) | 0.89 (0.83-0.94) | 0.86 (0.82-0.91) | 2.02 |
| 90 y and older | 13 923 | 1979 | 0.747 (0.736-0.758) | -0.070 (-0.082 to -0.059) | 0.94 (0.89-1.00) | 0.85 (0.80-0.90) | 2.08 |
| 80 y and older | 49 731 | 6521 | 0.752 (0.746-0.758) | -0.065 (-0.072 to -0.058) | 0.88 (0.85-0.91) | 0.88 (0.85-0.91) | 2.01 |

Abbreviations: CI, confidence interval; ICU, intensive care unit.

^a^The difference between an older band and the 18 to 64 y band uses the joint variance, as in Table 2.

## eTable 9. C Statistic by Anchor-Year Group

**Panel A. Stays by anchor-year group and age band, No.**

| Anchor-year group | 18 to 64 y | 65 to 74 y | 75 to 79 y | 80 to 84 y | 85 to 89 y | 90 y and older | 80 y and older |
| --- | --- | --- | --- | --- | --- | --- | --- |
| 2008-2010 | 73 746 | 20 114 | 7882 | 7104 | 6045 | 5679 | 18 828 |
| 2011-2013 | 80 828 | 15 172 | 5911 | 5391 | 4448 | 3402 | 13 241 |
| 2014-2016 | 76 924 | 13 710 | 4833 | 4239 | 3343 | 2819 | 10 401 |
| 2017-2019 | 55 642 | 9715 | 3817 | 2953 | 2285 | 2023 | 7261 |
| All groups | 287 140 | 58 711 | 22 443 | 19 687 | 16 121 | 13 923 | 49 731 |

**Panel B. C statistic (95% CI) by anchor-year group**

| Anchor-year group | 18 to 64 y | 80 y and older |
| --- | --- | --- |
| 2008-2010 | 0.795 (0.785-0.805) | 0.747 (0.736-0.759) |
| 2011-2013 | 0.812 (0.804-0.820) | 0.753 (0.742-0.764) |
| 2014-2016 | 0.826 (0.819-0.833) | 0.744 (0.732-0.756) |
| 2017-2019 | 0.834 (0.827-0.840) | 0.755 (0.744-0.767) |

Abbreviations: CI, confidence interval.

Anchor-year group is the patient-level de-identified calendar window in MIMIC-IV, not the encounter year.

## eTable 10. Ratio of Standard Errors With and Without Allowance for Repeat Stays by the Same Patient

**Panel A. C statistic by age band**

| Age band | Ratio^a^ |
| --- | --- |
| 18 to 64 y | 1.10 |
| 65 to 74 y | 1.08 |
| 75 to 79 y | 1.03 |
| 80 to 84 y | 1.02 |
| 85 to 89 y | 1.02 |
| 90 y and older | 1.03 |
| 80 y and older | 1.03 |

**Panel B. C statistic by anchor-year group**

| Anchor-year group | 18 to 64 y | 80 y and older |
| --- | --- | --- |
| 2008-2010 | 1.16 | 1.04 |
| 2011-2013 | 1.09 | 1.02 |
| 2014-2016 | 1.05 | 1.02 |
| 2017-2019 | 1.03 | 1.01 |

**Panel C. Under-triage age-band coefficients (vs 18 to 64 y)**

| Age band | Unadjusted | Adjusted, multiple imputation | Adjusted, complete case |
| --- | --- | --- | --- |
| **Six-level age-band coding** |  |  |  |
| 65 to 74 y | 1.04 | 1.02 | 1.02 |
| 75 to 79 y | 1.01 | 1.00 | 1.00 |
| 80 to 84 y | 1.02 | 1.01 | 1.02 |
| 85 to 89 y | 1.02 | 1.00 | 1.00 |
| 90 y and older | 1.02 | 1.00 | 1.00 |
| **Three-level age-band coding** |  |  |  |
| 65 to 79 y | 1.04 | 1.02 | 1.02 |
| 80 y and older | 1.03 | 1.01 | 1.01 |

^a^Standard error that allows for repeat stays by the same patient (patient-clustered for the C statistic, cluster-robust for the logistic models) divided by the standard error that treats stays as independent (independence or model-based). Anchor-year group is the patient-level de-identified calendar window in MIMIC-IV, not the encounter year.

## eTable 11. Missing Information: Bounds and Scenarios for Stays Without a Recorded ESI Level and for Transfers

**Panel A. Stays without a recorded ESI level: range of the C statistic by age band**

| Age band | Stays added | Added stays with the critical outcome | C statistic, primary analysis | Lowest possible | Highest possible |
| --- | --- | --- | --- | --- | --- |
| 18 to 64 y | 4482 | 1896 | 0.818 | 0.695 | 0.844 |
| 80 y and older | 1069 | 677 | 0.754 | 0.676 | 0.777 |

**Panel B. Transfers: range of the C statistic over the grid by age band^a^**

| Age band | Transfers counted as without the outcome | C statistic, primary analysis | Lowest | Highest |
| --- | --- | --- | --- | --- |
| 18 to 64 y | 1499 | 0.818 | 0.792 | 0.821 |
| 80 y and older | 438 | 0.754 | 0.731 | 0.754 |

**Panel C. Difference in C statistic, 80 y and older minus 18 to 64 y: scenarios and bounds**

| Analysis | C statistic, 80 y and older | C statistic, 18 to 64 y | Difference (95% CI) | Lower limit^b^ | Upper limit^b^ |
| --- | --- | --- | --- | --- | --- |
| Primary analysis | 0.754 | 0.818 | -0.065 (-0.072 to -0.058) |  |  |
| Stays without a recorded ESI level placed at ESI level 1 | 0.769 | 0.836 | -0.067 (-0.073 to -0.060) |  |  |
| Bounds over all ESI assignments to these stays |  |  |  | -0.167 | 0.082 |
| Every transfer counted as having the outcome | 0.732 | 0.795 | -0.063 (-0.071 to -0.056) |  |  |
| Bounds over the transfer grid |  |  |  | -0.089 | -0.037 |

Abbreviations: CI, confidence interval; ESI, Emergency Severity Index. The primary analysis uses stays with a recorded ESI level and counts transfers as without the outcome.

^a^Transfers reassigned as having the outcome in 10% steps per ESI level (0%, 10%, …, 100% of each level’s transfers), so the limits are extremes over that grid.

^b^Bounds carry no confidence interval.

## eTable 12. Chief-Complaint Category Against the First-Listed ED Diagnosis

| Chief-complaint category (matching ICD-10; ICD-9 codes) | 18 to 64 y: stays, No. | 18 to 64 y: matching/with diagnosis, No. (%) | 80 y and older: stays, No. | 80 y and older: matching/with diagnosis, No. (%) |
| --- | --- | --- | --- | --- |
| Trauma or injury (S00-T34; 800-959) | 36 686 | 23 301/36 600 (63.7) | 9223 | 5526/9185 (60.2) |
| Chest pain (R07, I20-I25; 786.5, 410-414) | 20 349 | 14 307/20 309 (70.4) | 3301 | 2222/3294 (67.5) |
| Dyspnea or respiratory (J00-J99, R06; 460-519, 786.0) | 14 647 | 8139/14 621 (55.7) | 6207 | 3041/6194 (49.1) |
| Fever or infection (A00-B99, R50; 001-139, 780.6) | 12 922 | 3363/12 891 (26.1) | 1307 | 411/1305 (31.5) |
| Psychiatric or substance (F00-F99, T36-T65; 290-319, 960-989) | 26 462 | 19 087/26 337 (72.5) | 420 | 177/419 (42.2) |

Abbreviations: ED, emergency department; ICD, International Classification of Diseases.

Matching is the number whose first-listed diagnosis falls in the ICD group of the category; the denominator is stays with a recorded first-listed ED diagnosis code, and the percentage is of that denominator. ICD-10 codes are listed before ICD-9 codes.

## eTable 13. ED Disposition, Deaths Within 7 and 30 Days of Arrival, and Deaths Not Counted in the Critical Outcome

**Panel A. ED disposition, with deaths within 7 and 30 days of arrival^a^**

| ED disposition | 18 to 64 y: stays, No. (%) | 18 to 64 y: death within 7 d, No. (%) | 18 to 64 y: death within 30 d, No. (%) | 80 y and older: stays, No. (%) | 80 y and older: death within 7 d, No. (%) | 80 y and older: death within 30 d, No. (%) |
| --- | --- | --- | --- | --- | --- | --- |
| Admitted (linked) | 110 093 (38.3) | 608 (0.6) | 1941 (1.8) | 36 452 (73.3) | 1143 (3.1) | 3227 (8.9) |
| Died in ED | 59 (<0.1) | 33 (55.9) | 33 (55.9) | 79 (0.2) | 71 (89.9) | 71 (89.9) |
| Home | 164 051 (57.1) | 30 (<0.1) | 135 (0.1) | 12 253 (24.6) | 35 (0.3) | 177 (1.4) |
| Left before completion | 10 274 (3.6) | 4 (<0.1) | 28 (0.3) | 383 (0.8) | 6 (1.6) | 11 (2.9) |
| Other or admitted without linked record | 1164 (0.4) | 3 (0.3) | 6 (0.5) | 126 (0.3) | 6 (4.8) | 10 (7.9) |
| Transfer | 1499 (0.5) | 2 (0.1) | 6 (0.4) | 438 (0.9) | 7 (1.6) | 29 (6.6) |
| All dispositions | 287 140 |  |  | 49 731 |  |  |

**Panel B. Stays without a linked admission and deaths within 7 days not counted in the critical outcome, by age band^b^**

| Age band | Without a linked admission, No. (%) | Counted in the critical outcome, No. | Death within 7 d, No. | Death within 7 d not counted in the critical outcome, No. | Not counted, % of stays with the critical outcome |
| --- | --- | --- | --- | --- | --- |
| 18 to 64 y | 177 047 (61.7) | 59 | 72 | 39 | 0.35 |
| 65 to 74 y | 22 497 (38.3) | 32 | 63 | 33 | 0.61 |
| 75 to 79 y | 7403 (33.0) | 14 | 27 | 15 | 0.61 |
| 80 to 84 y | 5653 (28.7) | 22 | 31 | 12 | 0.50 |
| 85 to 89 y | 4287 (26.6) | 19 | 38 | 21 | 1.02 |
| 90 y and older | 3339 (24.0) | 38 | 56 | 21 | 1.11 |
| 80 y and older | 13 279 (26.7) | 79 | 125 | 54 | 0.85 |

Abbreviations: ED, emergency department.

^a^Percentages of stays are of the band’s stays (287 140 and 49 731); percentages of deaths are of the stays in that row. Deaths are from the recorded date of death, counted from ED arrival. Stays are classified by linkage first, so an ED death with a linked admission record is counted under admitted, and “Died in ED” here (59 and 79) is smaller than ED deaths in Table 1.

^b^Percentages are of the band’s stays (Panel A and Table 1). Stays without a linked admission are those not admitted to a linked hospitalization. No outcome was reassigned.
